# Modelling TDP-43 proteinopathy in *Drosophila* uncovers shared and neuron-specific targets across ALS and FTD relevant circuits

**DOI:** 10.1186/s40478-023-01656-0

**Published:** 2023-10-20

**Authors:** R. Keating Godfrey, Eric Alsop, Reed T. Bjork, Brijesh S. Chauhan, Hillary C. Ruvalcaba, Jerry Antone, Lauren M. Gittings, Allison F. Michael, Christi Williams, Grace Hala’ufia, Alexander D. Blythe, Megan Hall, Rita Sattler, Kendall Van Keuren-Jensen, Daniela C. Zarnescu

**Affiliations:** 1 Department of Molecular and Cellular Biology, Life Sciences South, University of Arizona, 1007 E. Lowell St., Tucson, AZ 85721 USA; 2 Present Address: McGuire Center for Lepidoptera and Biodiversity, Florida Museum of Natural History, University of Florida, 3215 Hull Road, Gainesville, FL 32611 USA; 3 Translational Genomics Research Institute, 445 N 5th St., Phoenix, AZ 85004 USA; 4 Cellular and Molecular Physiology, Penn State College of Medicine, 500 University Drive Crescent Building C4605, Hershey, PA 17033 USA; 5 Department of Translational Neuroscience, Barrow Neurological Institute, 350 W Thomas Road, Phoenix, AZ 85013 USA

**Keywords:** ALS, FTD, TDP-43, Drosophila, Mushroom bodies, RNA-Seq, Glypican, Wnt signaling

## Abstract

Amyotrophic lateral sclerosis (ALS) and frontotemporal dementia (FTD) comprise a spectrum of neurodegenerative diseases linked to TDP-43 proteinopathy, which at the cellular level, is characterized by loss of nuclear TDP-43 and accumulation of cytoplasmic TDP-43 inclusions that ultimately cause RNA processing defects including dysregulation of splicing, mRNA transport and translation. Complementing our previous work in motor neurons, here we report a novel model of TDP-43 proteinopathy based on overexpression of TDP-43 in a subset of *Drosophila* Kenyon cells of the mushroom body (MB), a circuit with structural characteristics reminiscent of vertebrate cortical networks. This model recapitulates several aspects of dementia-relevant pathological features including age-dependent neuronal loss, nuclear depletion and cytoplasmic accumulation of TDP-43, and behavioral deficits in working memory and sleep that occur prior to axonal degeneration. RNA immunoprecipitations identify several candidate mRNA targets of TDP-43 in MBs, some of which are unique to the MB circuit and others that are shared with motor neurons. Among the latter is the glypican Dally-like-protein (Dlp), which exhibits significant TDP-43 associated reduction in expression during aging. Using genetic interactions we show that overexpression of Dlp in MBs mitigates TDP-43 dependent working memory deficits, conistent with Dlp acting as a mediator of TDP-43 toxicity. Substantiating our findings in the fly model, we find that the expression of *GPC6* mRNA, a human ortholog of *dlp,* is specifically altered in neurons exhibiting the molecular signature of TDP-43 pathology in FTD patient brains. These findings suggest that circuit-specific *Drosophila* models provide a platform for uncovering shared or disease-specific molecular mechanisms and vulnerabilities across the spectrum of TDP-43 proteinopathies.

**Supplementary Information:**

The online version contains supplementary material available at 10.1186/s40478-023-01656-0.

## Introduction

Characterized by extensive overlap in cellular pathology, genetic mutations, and molecular markers, amyotrophic lateral sclerosis (ALS) and frontotemporal dementia (FTD) comprise a neurodegenerative disease continuum affecting multiple subsets of neurons in the spinal cord, motor cortex, cingulate cortex, and frontal and temporal lobes [1–4]. Notably, nearly 97% of ALS cases [5] and approximately 45% of FTD cases [6] exhibit neurodegeneration with ubiquitin-positive cytoplasmic inclusions that contain transactive response (TAR) DNA binding protein 43 (TDP-43). A number of point mutations in *TARDBP*, the gene encoding TDP-43, have been identified as causative of ALS [7–9] while others have been linked to FTD [10, 11]. Although mutations in at least 20 other genes have been associated with ALS/FTD, the majority of ALS/FTD cases are sporadic [12].

Substantiating its significance as a marker of pathology, TDP-43 has also been identified as a component of cytoplasmic aggregates in dementias other than FTD, including Alzheimer’s disease (AD) [13–15], AD with Lewy body dementia (AD/LBD) [16], Parkinsonism-dementia complex (PDC) [17], and hippocampal sclerosis [18]. Up to 57% of AD cases exhibit TDP-43 positive intracellular inclusions [19]; notably, AD patients with TDP-43 proteinopathy show accelerated disease progression with more severe cognitive impairment [14, 20]. In addition to the presence of TDP-43 proteinopathy, dementias such as FTD and AD share TDP-43 associated cellular pathology, including dystrophic neurites [21]. Thus, despite differences in underlying genetic risk factors and specific neural populations affected, a large proportion of dementias exhibit overlapping TDP-43 pathology, providing a common entry point for understanding shared mechanisms of neurodegeneration [22, 23].

Wild-type TDP-43 is localized primarily to the nucleus where it regulates RNA transcription [24] and splicing [25–29] necessary for nervous system development and function. TDP-43 also shuttles between the nucleus and cytoplasm and has been shown to play a role in axonal and dendritic mRNA localization [30–32], stress granule dynamics [33–35] and translation [36–38]. During severe or prolonged cellular stress and in disease, TDP-43 exits the nucleus and associates with ubiquitin-positive cytoplasmic inclusions [39]. Indeed, cellular dysfunction and subsequent degeneration associated with TDP-43 pathology have been attributed to both loss-of-function of nuclear TDP-43 [27] and cytoplasmic gain-of-function [40] whereby cytoplasmic inclusions sequester mRNAs and perturb protein homeostasis [39].

Following the identification of TDP-43 as a protein of pathological interest in numerous neurodegenerative diseases, model organisms have been critical to understanding the normal biological functions of TDP-43 and how disruption of these functions leads to disease. Despite having diverged from a common ancestor with vertebrates over 600 million years ago [41], the fruit fly (*Drosophila melanogaster*) genome contains a homolog of *TARDBP*, namely *TBPH*, which displays sequence similarity and functional overlap with human TDP-43 [42, 43].

In addition to extensive overlap in neurogenetic homology, circuit-level conservation in the central nervous system has also been demonstrated between invertebrates and vertebrates [44–46], making invertebrate models relevant for understanding diseases of cognition. Indeed, flies display several behaviors affected in human disease [47] including associative learning [48], long-term memory [49], sleep [50], social interactions [51, 52], and addiction [53] that can serve as system-level readouts of molecular- and/or cellular-level dysfunction. Models of TDP-43 proteinopathy in *Drosophila* have recapitulated the locomotor phenotypes, motor neuron dysfunction, and shortened lifespan [43, 54, 55] observed in ALS and highlighted degeneration in central brain structures [54] reminiscent of FTD. Beyond simply simulating disease states, these fly models have identified translational targets [37, 38, 56] and genetic modifiers of TDP-43 proteinopathy [57–60], and exposed systemic effects on metabolic pathways [61, 62].

While TDP-43 proteinopathy has been extensively studied in motor neurons and glia as a model of ALS, dementia modeling based on TDP-43 proteinopathy in flies has been limited. This is in part because the mushroom bodies (MBs) of the fly brain, which were originally described as essential to odor learning [63] have only recently been recognized as being required for multi-modal and context-dependent learning [64, 65] including place learning [66]. The MBs also regulate sleep [67, 68], satiety [69], social behavior [70] and gate behaviors attributed to other brain regions such as decision-making [71] and aggression [72]. Therefore, the MBs share functional overlap with regions of the vertebrate cortex [73]. Existing fly models of intellectual disability [74] and Alzheimer’s disease (AD; [75] have either focused on, or identified phenotypes in the MBs of the fly brain, suggesting that this structure is appropriate for modeling cognitive disorders.

Here we describe the development of a novel fly model of dementia based on TDP-43 proteinopathy induced by specific over-expression of wild-type or mutant human TDP-43 in a well-defined subset of MB neurons. Our model recapitulates key cellular and behavioral characteristics of human dementias with TDP-43 pathology, including age-dependent loss of nuclear TDP-43, axonal degeneration, and working memory and sleep deficits that parallel those observed in dementia patients. Using RNA immunoprecipitations we show that in MBs, TDP-43 associates with several mRNAs, a subset of which are unique to MBs while others are shared with mRNA targets previously identified in motor neurons [38]. Interestingly, among the latter, we identified *dlp* mRNA, encoding the heparan sulfate proteoglycan (HSPG) Dally-like protein (Dlp)/GPC6, which we previously found to be a target of TDP-43 in fly models of ALS and is altered in human ALS spinal cords [38]. Here we report that *dlp* mRNA is enriched in TDP-43 complexes in MBs while Dlp protein is decreased within axons during aging, consistent with TDP-43 dependent axonal transport and/or translation deficits in an age-dependent manner. Notably, Dlp overexpression in MBs rescues TDP-43 dependent deficits in working memory consistent with Dlp being a physiologically significant target of TDP-43 in the MB circuit. These findings demonstrate that TDP-43 proteinopathy in MBs causes dementia-like phenotypes that are mediated at least in part by Dlp/GPC6, a regulator of the Wg/Wnt signaling pathway. Further substantiating the link between Dlp/GPC6 and TDP-43 pathology, we found *GPC6* mRNA to be altered in FTD patient brains. Specifically, neuronal nuclei expressing *STMN2* and *KALRN* cryptic exons associated with TDP-43 nuclear depletion show an upregulation of *GPC6* mRNA when compared with nuclei that retain TDP-43 as evidenced by the presence of canonically spliced *STMN2* and *KALRN* junctions. Lastly, we report a number of candidate targets of TDP-43 that show overlap between our fly models and patient brains, suggesting these models will be useful for studying molecular pathyways underlying distinct neuronal vulnerabilities across the spectrum of TDP-43 proteinopathies.

## Materials and methods

### Drosophila genetics and maintenance

Flies were maintained at 25 °C in 12-h light–dark cycle with 25–30% humidity. Specific information on *Drosophila* lines used in this study and specific experiments where each line was employed can be found in the Key Resources table (Additional file 1). For aging and lifespan studies, newly eclosed virgin male and female flies were collected and maintained on standard fly cornmeal/molasses media refreshed weekly. Flies harboring *UAS TDP-43::YFP* and *UAS dlp* transgenes for co-overexpression of TDP-43 and Dlp were generated using standard genetic recombination techniques. The presence of *UAS TDP-43::YFP* was confimed by YFP expression when crossed with the pan-neuronal driver, elav GAL4 while Dlp overexpression was confirmed using RT-qPCR (*dlp OE* = 2.45 FC, *TDP-43*^*WT*^*::YFP*; *dlp OE* = 3.09 FC, *TDP-43*^*G298S*^*::YFP*; *dlp OE* = 1.71 FC compared to *w*^*1118*^ controls; Additional file 1: Fig. S6-supplement 1b).

### Western blotting

One to three days old flies were collected from *SS01276* crossed with (1) *w*^*1118*^*; TDP-43*^*WT*^*::YFP*, (2) *w*^*1118*^*; TDP-43*^*WT*^*::YFP; dlp OE*, (3) *w*^*1118*^*; TDP-43*^*WT*^*::YFP mCD8::RFP*, and (4) *w*^*1118*^ in triplicate. Heads (N = 15 for each genotype) were decapitated and homogenized in 100 µl 2X Laemmli Sample Buffer (BIO-RAD 1610737) containing 5% of 2-Mercaptoethanol (Sigma-Aldrich M3148). The homogenized protein samples were boiled for 5 min in the digital Heat Block (Benchmark), and spun for 1 min. Supernatants were collected and 10 µl of protein sample was loaded in each well of precast Mini-PROTEAN TGX 4–20% gradient Gel (BIO-RAD 4561096). Following SDS-PAGE, the proteins were transferred to Nitrocellulose membrane (BIO-RAD 1620215). After transfer, the membrane was blocked in 5% nonfat milk in PBST (PBS and 0.1% TWEEN20) and incubated overnight at 4 °C with primary antibodies mouse anti-GFP Living Color (Cell Signaling Technology 2955, 1:1000) to detect TDP-43 YFP and rabbit anti-Beta-Actin (Cell Signaling Technology 4967S, 1:1000) as loading control, followed by washes in TBST and incubation with secondary antibody (IRDye® 800CW, Goat anti-Mouse D21115-25, 1:10,000) and (IRDye®680RD Goat anti-Rabbit D21207-05, 1:10,000) for 1 h at RT. The blot was imaged using a LICOR scanner (Odyssey®DLx) and protein bands intensities were quantified with LICOR “Image Studio Lite” (Additional file 1: Fig. S7-supplement 2).

### Statistical analyses

Statistical analyses were conducted in R [v. 4.1.2, R Core 76] and Rstudio [v. 2021.09.0 + 351, Rstudio 77] unless specified otherwise in methods. The tidyverse [78] and ggpubr [79] packages were used for summary statistics and graphics. Other analysis-specific packages are cited in the corresponding Methods section. While data for the mutant TDP-43^G298S^ model is presented as a supplement to our main findings, statistical analyses were conducted simultaneously for both TDP-43 genotypes and p-values were corrected for multiple comparisons that included both genotypes. For any given analysis when males and females did not differ statistically or sample sizes were low (*e.g.*, histological preparations), they were pooled to increase power. Where necessary, outliers were removed prior to hypothesis testing. When data did not fit the assumptions of parametric models and non-parametric analyses were used, hypothesis testing proceeded by first using a Kruskal-Wallace to test for a difference among groups, followed by pairwise comparisons using the Wilcoxon Rank Sum Test with p-values adjusted for multiple comparisons using the false discovery rate method [80]. Specific statistical methods are described for each assay. Summary statistics for each figure can be found in the Additional file 2.

### Mushroom body morphological analyses

Mushroom body morphology was evaluated using membrane-targeted RFP (mCD8 RFP) driven by *SS01276* [81]. Adult brains were dissected in cold HL-3 saline [82], fixed for 60 min in 4% paraformaldehyde, then rinsed in phosphate buffered saline (PBS, 3X), permeabilized in PBS with 0.25% Triton X-100 (PBST), and blocked in PBST plus 5% normal goat serum (Sigma-Aldrich 566,380) 2% bovine serum albumin (Sigma-Aldrich A5611) for 45 min prior to antibody labeling. YFP was detected by incubating brains overnight at 4 °C with a mouse monoclonal anti-GFP FITC antibody (1:300, Rockland 600-302-215). mCD8 RFP was visualized using native fluorescence. All brains were mounted on slides with the ventral side containing the mushroom body lobes (MBLs) facing the coverslip. For cell body imaging, brains were additionally incubated in Hoechst (1:10,000, Invitrogen H3570) for 10 min and mounted on slides with the dorsal side containing the calyx facing the coverslip.

### Image acquisition and analysis

TDP-43 cytoplasmic localization and MBN cell loss: Images were acquired using a Zeiss 880 Laser Scanning Confocal inverted microscope with a Plan-Apochromat 63x/1.4 oil DIC M27 lens. TDP-43 YFP signal intensity was quantified per unit area in the nucleus and cell body. Hoechst was used to define the nuclear boundaries, while total cellular TDP-43 signal was measured by tracing a boundary around YFP signal in the entire cell (Additional file 1: Fig. S1-supplement 3). To quantify the total number of MBNs expressing TDP-43 YFP, all nuclei were counted from optical sections 2 μm apart to ensure nuclei were counted only once. We tested for differences in TDP-43 nuclear to cellular ratio and MBN number by age, sex, and genotype using Analysis of Variance (ANOVA) performed on a linear model (*lm* function in R; R Core Team 2017). Residuals were normally distributed (Shapiro–Wilks test for normality, *P* = 0.890 and *P* = 0.395 for ratio and MBN number, respectively) and we therefore continued with pairwise comparisons using Tukey’s Honest Significant Difference corrected for multiple comparisons.

Signal intensity: All images were acquired using a Zeiss 880 Laser Scanning Confocal inverted microscope with a Plan-Apochromat 63×/1.4 oil DIC M27 lens. For mCD8 RFP fluorescence intensity, brains were imaged using either an Alexa Fluor 568 or a DS Red filter set with pinhole adjusted to 1 AU and 3 µm optical section thickness. For measuring anti-GFP FITC fluorescence intensity, brains were imaged using the FITC filter set with the pinhole adjusted to 1 AU and 0.5 µm optical section thickness. Fluorescence intensity was measured in FIJI as the integrated density over the sample area in axons by manually tracing α/β or γ lobes and subtracting adjacent background signal. First, a polygon was traced free-hand over the visible area of each lobe (α/β or g). This polygon was then dragged to an adjacent area of the brain to obtain a mean fluorescence intensity for the background in each section. In instances where the polygon was shaped in such a way that it could not be dragged to adjacent brain area, a comparably-sized polygon was drawn in the adjacent brain region. Intensity was measured in at least three sections to obtain mean fluorescence intensity in each channel (RFP or FITC) for both α/β and γ lobes of each brain. To test for differences in signal intensity within a genotype across age time points, values of signal intensity were normalized to mean signal intensity of young (1–3 days old) brains of the same genotype and lobe. Statistical analysis of change in fluorescence intensity with age was performed by comparing middle aged (~ 30 days) or old (~ 60 days) flies and young (~ 1 day) flies from the same genotype using the Wilcoxon Rank Sum test. Summary statistics can be found in Additional file 2: Table S2a.

YFP particle size: The Analyze Particles function in FIJI was used to identify YFP puncta in MB lobes. Images were first contrast enhanced using a brightness/contrast enhancement (saturated pixels set to 0.3% and normalized) followed by thresholding using the Bernsen method in the Auto Local Threshold function with a 25–50 pixel radius depending on image quality. Particles were considered puncta when they were 0.25–3.0 µm^2^ in size and showed 0.25–1.0 circularity. To measure mean particle size in each sample, a polygon was drawn over the visible portion of each lobe in at least three sections for each lobe. From these subsamples we calculated a mean particle size for each lobe of each brain. Summary statistics can be found in Additional file 2: Table S2B.

Dally-like protein expression during aging in MBs: Brains from flies 1–3 days or 50–55 days were dissected out and the tissue fixed, permeabilized and blocked as described above. Brains were then incubated overnight at 4 °C in an anti-Dally-like protein antibody at 1:5 in block (13G8 developed by Phil Beachy, obtained from the Developmental Studies Hybridoma Bank, created by the NICHD of the NIH and maintained at The University of Iowa, Department of Biology, Iowa City, IA 52242, targets amino acids V523 to Q702), rinsed 3X in 0.1% PBST and incubated in goat anti-mouse Alexa Flour 647 antibody overnight at 4 °C (1:300, ThermoFisher A32728). Images were acquired using a Zeiss 880 Laser Scanning Confocal inverted microscope with a Plan-Apochromat 63×/1.4 oil DIC M27 lens. Signal intensity was traced from maximum intensity projections of the MBLs and normalized to background intensity. Dlp signal intensity declined with age in TDP-43 flies, so the mCD8 RFP channel was used to trace the lobes in aged brains, ensuring the correct lobe area was used for signal intensity measurements.

GAL4 dilution effect: To test whether UAS-driven expression of a second transgene reduces UAS-driven TDP-43^WT^ expression, in addition to western blots, we also measured mean pixel intensity from maximum intensity projections of the horizontal lobes (β and γ combined in maximum intensity projection) in two genotypes with *SS01276*-driven transgenes, *OR-R;TDP-43*^*WT*^*::YFP* and *w*^*1118*^*; TDP-43*^*WT*^*::YFP mCD8::RFP* (Additional file 1: Fig. S7-supplment 3).

### Behavioral assays

Y-maze: To measure changes in working memory, we employed a y-maze assay described by Lewis, Negelspach, Kaladchibachi, Cowen and Fernandez [83]. We tracked the two-dimensional movement of individual *Drosophila* placed in small symmetrical y-mazes using video recordings for 10 one-minute trials following two minutes of acclimation time in the maze. Observations took place between 08:45 and 11:00 and 14:00 to 16:00 when flies were most active. Male and female flies were run separately and the locations of genotypes in the seven by seven maze array were randomized for each set of trials. Assays were conducted in a dark room with each maze lit uniformly from below with a white LED array and capped with a clear Plexiglas coverslip. The overall movement of flies and visits to three unique arms consecutively were quantified using the Noldus Ethovision software. Spontaneous alternation behavior was measured by scoring three consecutive arm entries in a sliding window for each of ten trials. The alternation score was calculated as alternations divided by alternation attempts. Movement and alternation data were summed for each fly during each one-minute trial and then averaged over 10 trials for statistical analyses. Flies originating from three biological replicates were pooled for analysis (Additional file 2: Table S3). The large samples sizes permitted the use of parametric tests, and we assessed differences in distance moved or alternation score across genotypes using ANOVA on a linear mixed-effects model [lmer function in R; 84] that included replicate as a fixed effect. Flies that performed no alternations naturally did not have a percent alternation score and were removed from subsequent analysis. Males and females were analyzed separately. Flies were additionally assessed for the presence of bias for specific arms, calculated as a ratio of the mean number of entries into each arm over mean total entries and tested for significant variation from 0.333 using the Wilcoxon Signed Rank test. Interestingly, while females showed no arm biases, males overexpressing *TDP-43*^*WT*^ and OR-R controls showed a bias against arm B (*TDP-43*^*WT*^, $\overline{x }$ = 0.307 ± $0.08$, *P* < 0.0001; OR-R, $\overline{x }$ = 0.316 ± 0.13, *P* = 0.0038). *TDP-43*^*WT*^ also showed a bias for arm A ($\overline{x }$ = 0.352 ± 0.076, *P* = 0.0033), while OR-R controls showed a bias for arm C ($\overline{x }$ = 0.347 ± 0.113, *P* = 0.048). Although these analyses suggest that arm biases exist in males and are not unique to TDP-43 overexpression, a single replicate was used to test whether Dlp over-expression could rescue alternation deficits seen in TDP-43 overexpressing flies therefore hypothesis testing proceeded with non-parametric tests as described in *Statistical analyses*.

Sleep: Adult flies from the three age time points were monitored individually using *Drosophila* Activity Monitors [DAMs; Trikinetics, Waltham, MA; 85]. Flies were placed in monitoring tubes with a small amount of food the day before they reached two to three, 31–33, or 61–63 days old. Sleep and activity monitoring began at 12 AM following placement of flies in the sleep incubator, allowing flies at least six hours prior to the start of the experiment to acclimate to the tubes. Sleep and locomotor activity data were collected at one-minute intervals for three days and analyzed using ShinyR-DAM [86]. In *Drosophila,* sleep is defined at five minutes of inactivity [50] and here sleep data were processed using the ShinyR DAM program (Cichewicz and Hirsh 2018), which provided average sleep, sleep bout number and duration, and activity to sleep bout length ratio. Comparison of these variables by age and genotype were conducted separately for male and female flies. Data from two replicates were pooled (> 15 flies per genotype × replicate; sample sizes in Additional file 2: Tables S4A and S4B).

### Lifespan

Newly eclosed virgin males and females were separated and placed on fresh food, then transferred weekly into new food vials. The number of surviving flies was counted every other day for 100 days. Survival analysis and plots were generated using R packages *survival* [87] and *survivminer* [88].

### mRNA targets of TDP-43

RNA Immunoprecipitations: Equal numbers of male and female flies, aged one to three days, were pooled in Eppendorf tubes and flash frozen in liquid nitrogen. A minimum of 500 flies expressing YFP, TDP-43^WT^ YFP, or TDP-43^G298S^ YFP were collected for each of three biological replicates. Frozen flies were then transferred to 50 mL conical tubes and heads were separated from the bodies using four rounds of vortexing and flash freezing with liquid nitrogen. A sieve was used to filter out the bodies and isolated heads were collected into tubes containing lysis beads (Next Advance Green lysis beads). Heads were homogenized (Next Advance Bullet Blender) in one mL fresh lysis buffer (DEPC water, 50 mM HEPES buffer pH 7.4, 0.5% Triton X-100, 150 mM NaCl, 30 mM EDTA), protease inhibitors (cOmplete™, Mini, EDTA-free Protease Inhibitor Cocktail, Millipore Sigma 11873580001), and RNAsin Plus 400 u/mL (Fischer Scientific PRN2615), centrifuged for 10 min at 10,000 rpm, and the lysate was collected. A portion of the lysate was saved for the protein input and RNA input samples. For protein input, the lysate was mixed with 2X Laemmli buffer, boiled at 95–100 °C for 10 min, and stored at − 20 °C. For RNA input, TRIzol reagent (Thermofisher 15596026) was added to the lysate and the sample was stored at − 80 °C. High affinity GFP-Trap magnetic agarose beads (ChromoTek) were added to the remaining lysate and rotated end-over-end for 90 min at 4 °C to allow for binding of YFP. The beads were separated from the lysate with a magnet and washed three times with fresh wash buffer (DEPC water, 50 mM HEPES buffer pH 7.4, 0.5% Triton X-100, 150 mM NaCl, 30 mM EDTA). The beads were resuspended in wash buffer and split into two tubes for the protein IP and RNA IP samples. For the protein IP sample, 2X Laemmli buffer was added to the beads, samples were boiled at 95–100 °C for 10 min, and the beads were removed with a magnet. Western blots (see above) were performed to ensure that the immunoprecipitated complexes contained TDP-43 before processing the RNA IPs. For the RNA IP sample, TRIzol reagent was added to the beads, the solution was pipetted up and down for 60 s, and the beads were removed with a magnet.

RNA-Seq: RNA was quantified by nanodrop to bring it within range for ribogreen quantification. RNA was also checked using Agilent Tapestation High Sensitivitiy RNA screentape. One ng total RNA was used for SMART-Seq HT PLUS (Takara Bio USA, Inc. Cat # R400748) following manufacturer’s protocol. Determination of cDNA quality and quantity was determined via Agilent Tapestation High Sensitivity D5000 Screentape and Qubit dsDNA HS assay for input into library amplification. Libraries were quantified by Agilent Tapestation High Sensitivity D1000 Screentape and Kapa Library Quantification kit for Illumina platforms. Libraries were pooled equimolarly, pools were quantified by Agilent Tapestation High Sensitivity D1000 Screentape and Kapa Library Quantification kit and loaded on the NovaSeq 6000 S4 flowcell and sequenced to 101 × 11 × 11 × 101 cycles. Trimmed fastqs were aligned to the Dmel genome with STAR v2.6.1d [89]. Aligned reads were counted with featureCounts v1.6.3 [90] using the genome annotation. Files produced for individual samples were aggregated into a single.txt file using python. Differential expression was quantified using Deseq2 [91]. We included over-expression of cytoplasmic YFP in MBNs as a control for IPs, however the expression levels of YFP alone were far greater than cytoplasmic TDP-43 YFP (data not shown), making it difficult to assess the role of cytoplasmic YFP alone. To ensure that candidate mRNA targets showing the greatest enrichment were indeed specific in their association with TDP-43, we subtracted YFP control Log2FC values from the Log2FC values of targets recovered in each of our models. Indeed, in this YFP-subtracted analysis *futsch* was recovered as enriched with TDP-43 in both variants (Fig. 6a, Additional file 1: Fig. S6-supplment 1a) and the majority of mRNAs that showed high enrichment in the original analysis were retained in the YFP-subtracted analyses (Fig. 6a vs 6d, Additional file 1: Fig. S6A vs S6C).

Functional annotation: The Database for Annotation, Visualization, and Integrated Discovery (DAVID) was used to functionally annotated genes enriched with TDP-43 [92, 93]. To assess genes over-represented in our disease model data sets, we used lists of genes significantly enriched with each of our TDP-43 models (differential gene expression lists available in source data).

RNA Extraction for Validation: RNA input and RNA IP samples, stored in TRIzol reagent (Thermofisher 15596026) at − 80 °C, were allowed to thaw completely on ice and chloroform was added to each sample. Samples were shaken briefly to mix, left to incubate for three minutes at room temperature, and then centrifuged at 12,000 rpm for 15 minutes at 4 °C to allow for separation of the aqueous and organic phases. The RNA-containing aqueous phase was collected and molecular grade isopropyl alcohol was added to each sample. Samples were incubated for 10 min at room temperature for RNA precipitation and then centrifuged at 12,000 rpm for 10 minutes. The pelleted RNA was washed with 75% ethanol (200 proof ethanol & HyPure water) and left to dry in a fume hood. RNA was resuspended in HyPure water and the concentration and 260/280 absorbance ratio were measured using Nanodrop.

RT-qPCR: RNA extracted from input and IP samples was used as template RNA for cDNA synthesis by reverse transcription. Total RNA used in the cDNA synthesis reactions was normalized across samples. cDNA was synthesized using the Fisher First Strand cDNA synthesis kit (Thermofisher Scientific K1641). qPCR reactions were prepared in a 96-well qPCR plate, in triplicates, using Taqman Fast Advanced Master Mix (Thermofisher Scientific 4444556) and Taqman probes for Dally-like protein (dlp) (Thermofisher Scientific Dm01798597_m1) and Gpdh1 (Thermofisher Scientific Dm01841185_m1). qPCR was conducted on the qTOWER (Analytik Jena 844-00504-4) qPCR machine. Delta Ct was calculated as the difference in Ct values (dlp—gpdh1). To determine enrichment of dlp mRNA in the IPs, ΔΔCt was calculated as the difference in Delta Ct values (IP—Input). Fold change was calculated as 2^(− ΔΔCt).

Dally-like protein target validation: Brains from flies one to three days or 50–55 days were dissected out and the tissue fixed, permeabilized and blocked as described above. Brains were then incubated overnight at 4 °C in an anti-Dally-like protein antibody at 1:5 in block (13G8 developed by Phil Beachy, obtained from the Developmental Studies Hybridoma Bank, created by the NICHD of the NIH and maintained at The University of Iowa, Department of Biology, Iowa City, IA 52242, targets amino acids V523 to Q702), rinsed 3X in 0.1% PBST and incubated in goat anti-mouse Alexa Flour 647 antibody overnight at 4 °C (1:300, ThermoFisher A32728). Images were acquired using a Zeiss 880 Laser Scanning Confocal inverted microscope with a Plan-Apochromat 63×/1.4 oil DIC M27 lens. Signal intensity was traced from maximum intensity projections of the MBLs and normalized to background intensity. Dlp signal intensity declined with age in TDP-43 flies, so the mCD8 RFP channel was used to trace the lobes in aged brains, ensuring the correct lobe area was used for signal intensity measurements.

snRNA seq analyses from FTD patient brains: Log normalized gene counts tables from [94] were filtered to neuronal nuclei containing either a *STMN2* or *KALRN* cryptic exon or a *STMN2* or *KALRN* canonical junction, as defined in [94]. Human orthologs of Drosophila genes were identified using the DIOPT tool [95]. *GPC6* gene expression was compared between neuronal nuclei containing *STMN2* and *KALRN* cryptic exons and neuronal nuclei containing *STMN2* and *KALRN* canonical splice junctions using the independent t-test function (ttest_ind) in python. Violin plots were generated between neuronal nuclei containing a cryptic exon and neuronal nuclei containing a canonical exon using the scanpy violin function.

## Results

### Overexpression of TDP-43 in Kenyon cells results in age-dependent cell loss and nuclear to cytoplasmic mislocalization

The insect MBs are comprised of large, complex neurons called Kenyon cells, referred herein as MB neurons (MBNs), which form a layered network with reticular feedback [96], structural characteristics reminiscent of vertebrate cortical neurons and networks. There are three subtypes of MBNs based on developmental origin, cell morphology, and wiring. Axons of these three types form five layered lobes, the α/β, α′/β′, and γ lobes, which are further compartmentalized into columnar-like regions based on neuromodulatory inputs and excitatory or inhibitory post synaptic partners [97]. To model FTD-like TDP-43 proteinopathy we used a highly specific split GAL4 driver line *SS01276* available from the Janelia FlyLight Split-GAL4 Driver Collection [81] to express human TDP-43 [98] in a subset of MBNs that form the α/β and γ lobes of the adult *Drosophila* MBs. Previous studies have shown that neurons forming the γ and α′/β′ lobes are embryonic and larval in origin, respectively [99], and persist through metamorphosis, whereas those comprising the α/β lobes are born after puparium formation [100].

**Fig. 1:**
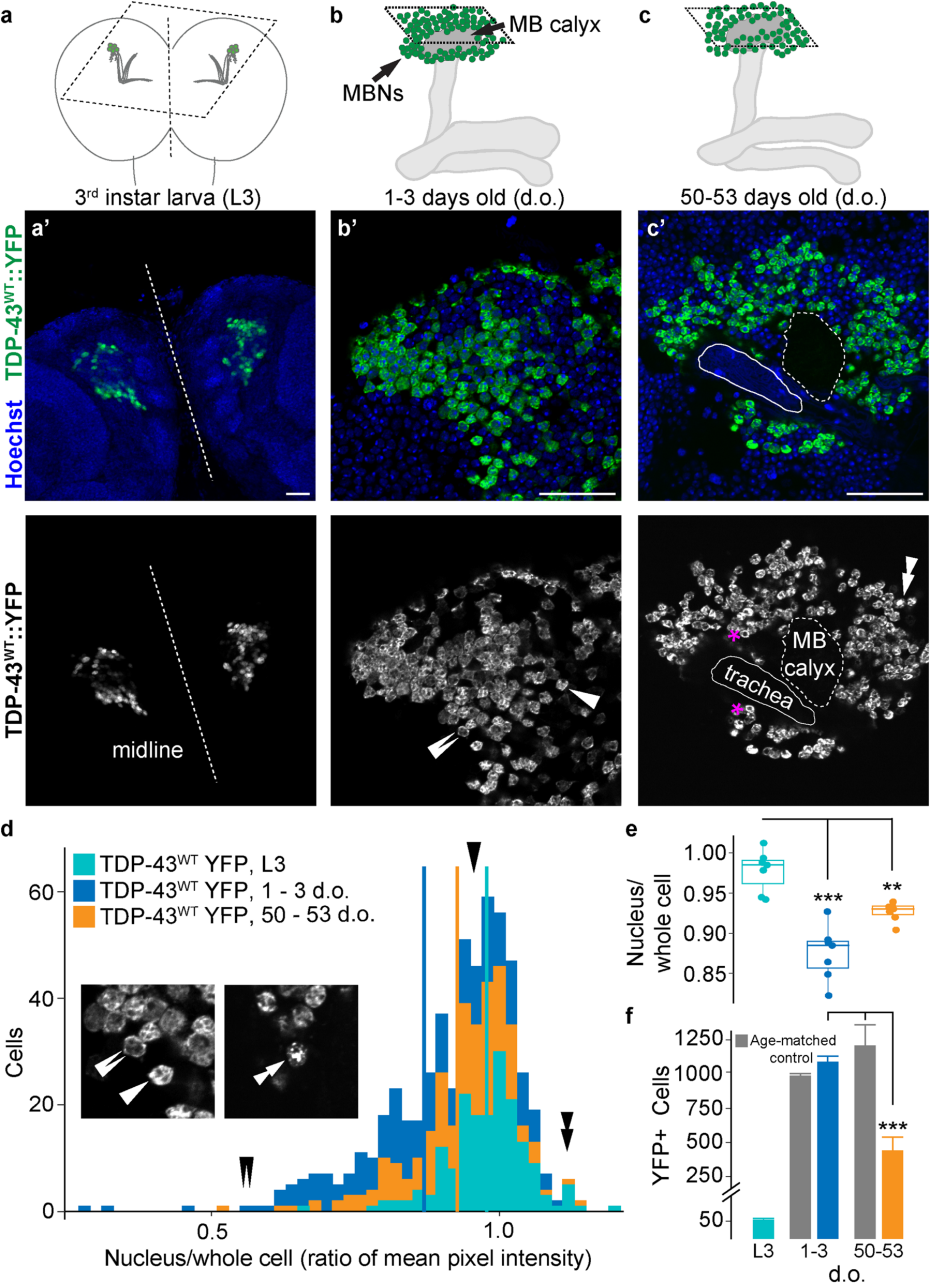
*TDP-43*^*WT*^ overexpression in Kenyon cells results in cytoplasmic mis-localization and age-dependent cell loss. Illustrations in **a**, **b**, and **c** correspond to approximate plane of imaging for Kenyon cells (MBNs) in **a’** larval, **b’** young adult, and **c’** old adult brain. **a’** Nuclear localization of human TDP-43^WT^ YFP in the larval MBNs. **b’** Young adult flies (1–3 days) contain cells with TDP-43^WT^ YFP in the cell body and cells showing nuclear loss of TDP-43^WT^ YFP (adjacent, double arrows as compared with single arrow). **c’** Old adults (50–55 days) have fewer cells with nuclear depletion but concurrent loss in total number of TDP-43^WT^ positive neurons (quantified in **e** and **f**). Stacked, double arrows indicate a cell that has a signal intensity ratio > 1.0 due to punctate TDP-43 signal in the nucleus. Magenta asterisks indicate cells where nuclear depletion can be detected visually. **d** Histograms showing distribution of TDP-43^WT^ YFP signal intensity ratios (cell nuclei to whole cell) shift with age. Arrows correspond to measurements taken from cells shown in figure inset taken from **b’**, **c’**. **e** Age-specific TDP-43^WT^ YFP signal intensity ratios. **f** Decrease in TDP-43^WT^ YFP positive cells from young to old adults. *SS01276* > *YFP* used as age-matched controls. Larval cell numbers provided for reference, but not used in statistical comparisons. Data for TDP-43^G298S^ YFP presented in Fig. 1-supplement 2. Scale bars = 50 µm. ** = *P* < 0.01; *** = *P* < 0.001

We characterized expression driven by *SS01276* in the 3rd instar larva (L3) and found that cytoplasmic YFP, YFP tagged TDP-43 (*TDP-43::YFP*) and membrane targeted RFP (*mCD8:: RFP*) are limited to MBNs (Additional file 1: Fig. S1-supplement 1). Signal is detected in approximately 50 larval MBNs in both TDP-43^WT^ overexpression (OE) and TDP-43^G298S^ OE brains (Fig. 1a, f *TDP-43*^*WT*^*::YFP mCD8::RFP*; Additional file 1: Fig. S1-supplement 2, *TDP-43*^*G298S*^*::YFP mCD8::RFP*). In L3, the MBs comprise approximately 60 γ lobe MBNs and 40 α′/β′ lobe MBNs [100] thus at this stage it is formally possible that *SS01276* drives expression in some combination of γ lobe or α′/β′ lobe MBNs. However, since adult expression of *SS01276* was determined to be limited to γ and α/β lobe MBNs [81], and given that both γ and α′/β′ lobe MBNs persist through metamorphosis, it is likely that the expression we observed during L3 for *SS01276* corresponds to γ lobe MBNs only.

Visual inspection of larval MBNs indicated that TDP-43 YFP was largely confined to the nucleus in L3 brains (Fig. 1a) but became mislocalized outside the nucleus in young adults (Fig. 1b). The nucleus comprises nearly all of the cell body in MBNs, making it challenging to accurately assess cytoplasmic mislocalization or nuclear depletion. Therefore, to determine age-dependent changes in TDP-43 localization, we measured mean signal intensity of TDP-43 YFP in the nucleus by tracing the nucleus in the Hoechst channel, then measured mean signal intensity of TDP-43 YFP in the entire cell by tracing the YFP signal of the entire cell boundary (see Materials and Methods and Additional file 1: Fig. S1-supplement 3) and calculated the ratio of nucleus to total cell signal mean intensity (Fig. 1d, e). Since the MBN cytoplasms are extremely thin, this ratio should be nearly 1.0 when TDP-43 is localized to both the nucleus and the cytoplasm (single arrow, Figs. 1b, d), less than 1.0 when TDP-43 YFP is depleted from the nucleus and mislocalized to the cytoplasm (see double arrows, Fig. 1b, d) and slightly above 1.0 when TDP-43 is contained largely in the nucleus (see stacked arrows, Fig. 1c, d).

Using this measure of nuclear depletion, we found a significant decrease in the mean signal intensity ratio across the population of MBNs from juvenile to young adults in the context of both TDP-43 OE variants (Fig. 1e, *TDP-43*^*WT*^*::YFP mCD8::RFP* 0.978 $\pm$ 0.005 in L3 vs. 0.869 $\pm$ 0.009 in young adults, *P* < 0.0001; Additional file 1: Fig. S1-supplement 2, *TDP-43*^*G298S*^*:: YFP mCD8::RFP* 0.967 $\pm$ 0.002 in L3 vs 0.906 $\pm$ 0.012 in young adults, *P* = 0.0004; Additional file 2: Table S1E). Surprisingly, the ratio of nucleus to total cell signal mean intensity increased in old adult brains compared to young adults, however, it was still significantly lower than at the juvenile age time point (L3) in our TDP-43^WT^ OE model (Fig. 1e, 0.978 $\pm$ 0.005 in L3 vs. 0.927 $\pm$ 0.009 in old adults, *P* = 0.0041; Additional file 2: Table S1E). A plausible explanation for this result is provided by our findings that there are significantly fewer TDP-43 YFP positive MBNs in old compared to younger TDP-43 OE brains or to old YFP controls (*TDP-43*^*WT*^*:: YFP mCD8::RFP,* 445 $\pm$ 96 cells in old adults vs 1089 $\pm$ 40 cells in young adults vs. 1206 $\pm$ 150 cells in YFP old adults, *P* < 0.001; Fig. 1f). Similar results were obtained for TDP^G298S^ YFP expressing brains (*TDP-43*^*G298S*^*:: YFP mCD8::RFP* 473 $\pm$ 79 cells in old adults vs 1097 $\pm$ 55 cells in young adults vs 1206 $\pm$ 150 cells in old adults expressing YFP only, *P* < 0.001; Additional file 1: Fig. S1-supplemnt 2; Additional file 2: Table S1f). Although more work is needed to show that neurons with reduced nuclear TDP-43 are selectively dying, these results show that TDP-43 OE causing age-dependent cell loss and are consistent with a scenario in which MBNs exhibiting increased levels of cytoplasmic TDP-43 are more vulnerable and lost during aging, likely through increased cell death.

### Mushroom body axons exhibit age-dependent TDP-43 puncta accumulation and progressive degeneration

**Fig. 2:**
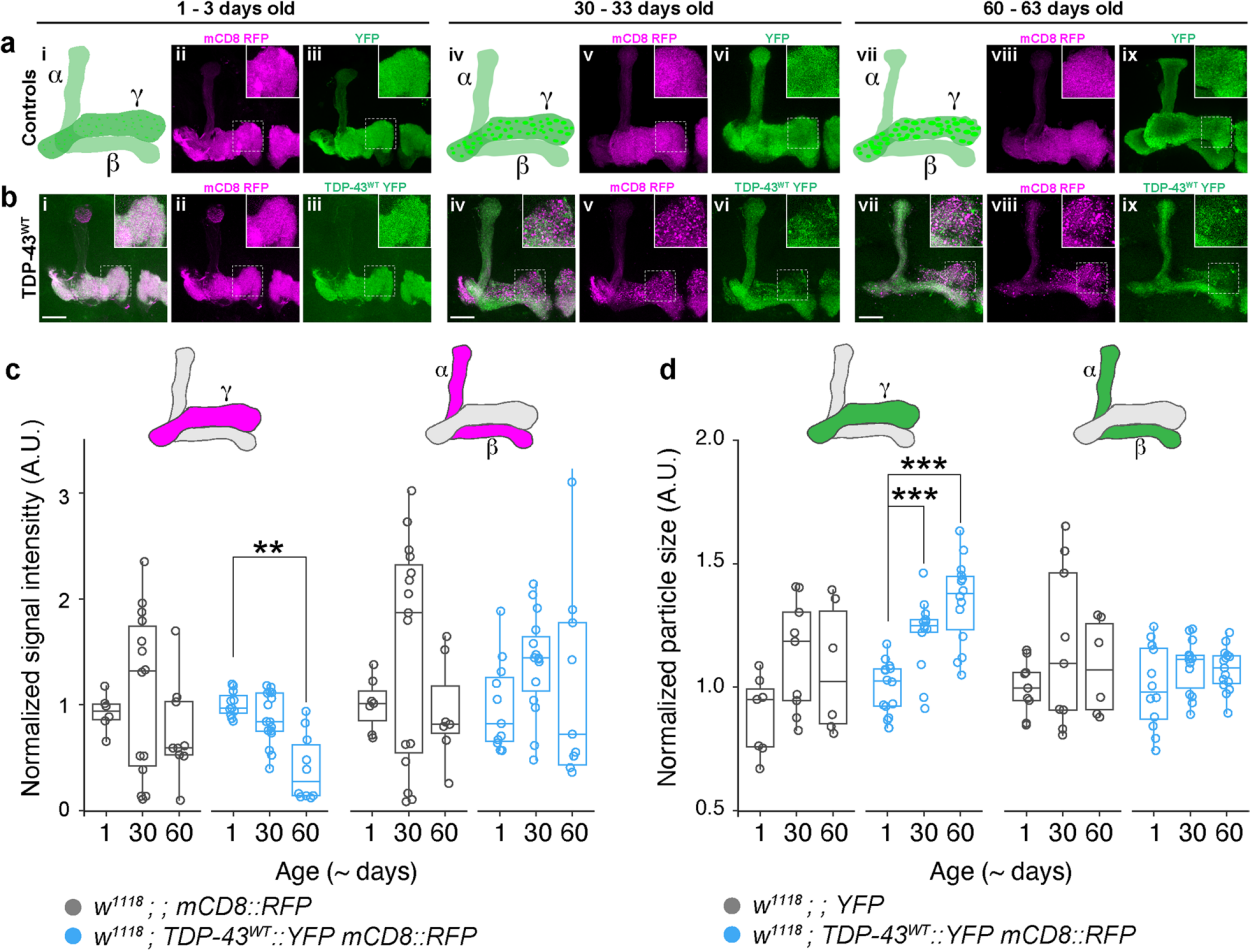
Mushroom body lobes (MBLs) show age-related, region-specific *TDP-43*^*WT*^ accumulation and axonal fragmentation. **a** Illustrations of MBLs targeted by *TDP-43*^*WT*^ OE and observed changes with age (i, intact in young flies; iv, increasingly punctate TDP-43 YFP by middle-age; vii, axonal thinning and evidence of degeneration specifically in the γ lobe). These are presented alongside morphology of young (ii, iii,) middle-aged (v, vi) and old (viii, ix) control flies expressing membrane-bound RFP (mCD8 RFP) or cytoplasmic YFP. **b** Over expression of TDP-43^WT^ YFP in MBNs results in axonal localization of TDP-43 in young adult flies (iii) and dystrophic neurites in middle-aged (v) and old (viii) flies. **c** Signal intensity of mCD8 RFP with age in γ (left) and α/β (right) lobes. **d** Changes in TDP-43^WT^ YFP particle size with age in γ (left) and α/β (right) lobes. Data for TDP-43^G298S^ presented in Fig. 2-supplement 1. Scale bar = 25 µm * = *P* < 0.05; ** = *P* < 0.01; *** = *P* < 0.001

To determine the effect of TDP-43 proteinopathy on axonal integrity and overall lobe morphology, we co-expressed TDP-43 and the membrane marker mCD8 RFP. Signal intensity of mCD8 RFP showed progressive decrease in the γ lobe over time (young, 1.01 $\pm$ 0.04, middle-aged, 0.87 $\pm$ 0.25, and old, 0.40 $\pm$ 0.10 fluorescence intensity ratio to age matched controls; Fig. 2a, b, insets b, e, h; quantified in c; Additional file 2: Table S2a), suggesting that axonal thinning occurred in an age-dependent manner. Simultaneously, TDP-43 formed increasingly larger axonal puncta in the γ lobe with age (young, 1.00 $\pm$ 0.04, middle-aged, 1.19 $\pm$ 0.05, and old, 1.35 $\pm$ 0.05 area normalized to age matched controls Fig. 2b, insets iii, vi, ix; quantified in d; Additional file 2: Table S2B). These findings are consistent with the formation of dystrophic axons, membrane fragmentation and cytoplasmic aggregation of TDP-43. A similar pattern of axonal loss and fragmentation in the γ lobe, coincident with increased TDP-43 puncta size, was observed in TDP-43^G298S^ OE flies (Additional file 1: Fig. S1-supplement 1a, b insets iii, vi, ix, quantified in c and d; Additional file 2: Tables S2a and b). We could not detect statistically significant changes in mCD8 RFP signal intensity nor TDP-43 cytoplasmic puncta in the α/β lobes of TDP-43^WT^ OE flies, however these lobes showed dramatic, age-dependent thinning not observed in controls (Fig. 2b inset i). In TDP-43^G298S^ OE brains, we observed both an increase in the size of TDP-43 YFP cytoplasmic puncta within α/β lobes between young and middle-aged adults, and a thinning of these lobes (Additional file 1: Fig. S2-supplement 1b inset i). Interestingly, these phenotypes are reminiscent of the age-dependent pathophysiology observed in human patients. Given that γ lobe neurons are born earlier than those within the α/β lobes, the more severe phenotypes observed in this neuronal subpopulation highlight the potential contribution of aging to TDP-43 proteinopathy.

### TDP-43 proteinopathy causes working memory deficits

**Fig. 3:**
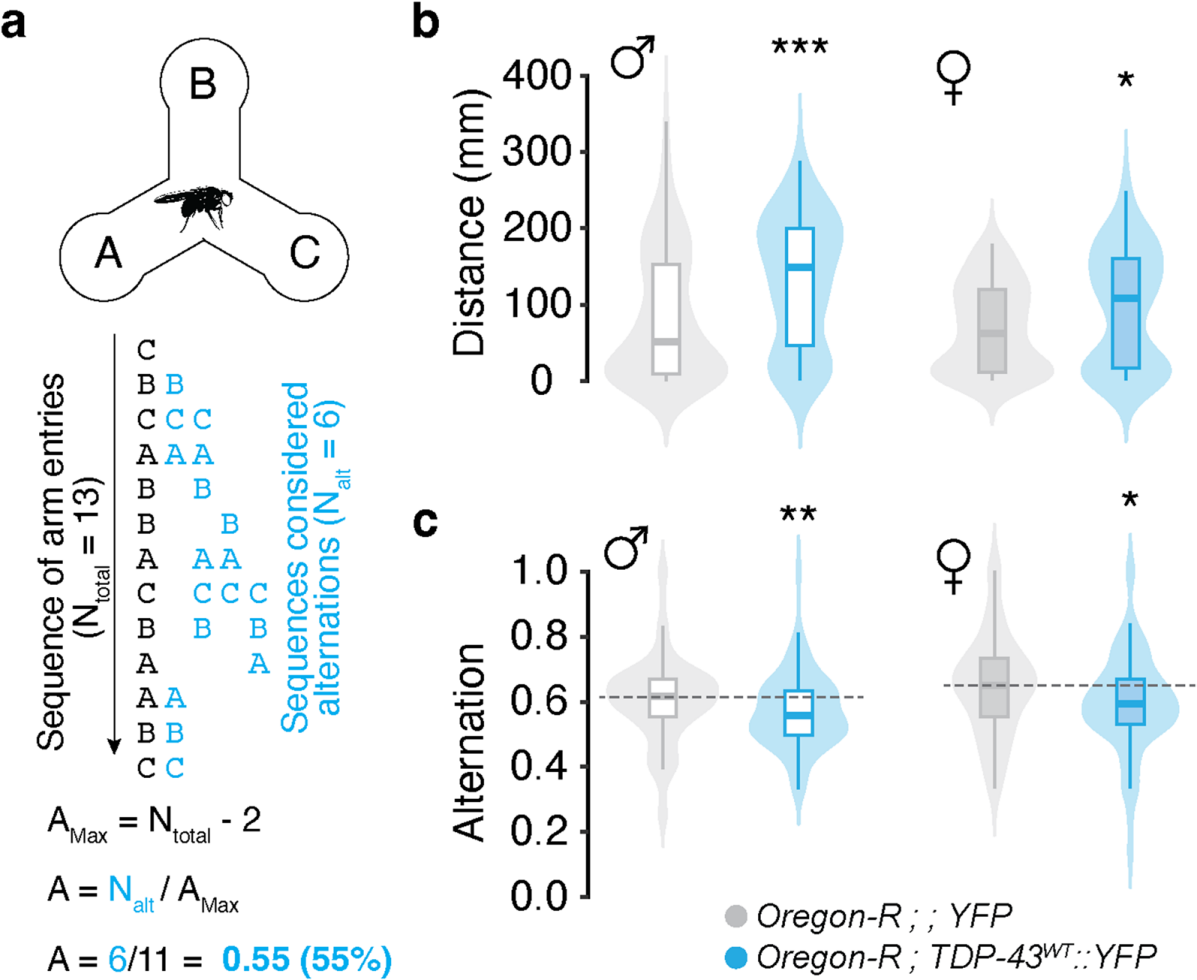
Overexpression of TDP-43 in MBNs results in spatial working memory deficits. **a** Schematic of the spatial working memory assay showing the sequence of decisions made by a fly (black text) and those coded as alternations (blue text). Alternation scores were calculated as the number of alternations (N_alt_) divided by alternation attempts (A_max_). **b** Young (1–3 days old) flies with TDP-43^WT^ YFP OE show significant increases in movement. **c** Despite moving more, these flies show reduced alternation percent when compared with controls. Grey dotted line = control group median. A% = alternation percent. * = *P* < 0.05; ** = *P* < 0.01; *** = *P* < 0.001

In humans, working memory is disrupted in FTD and other TDP-43 associated dementias [101, 102]. To test whether flies overexpressing TDP-43 exhibit working memory deficits, we used miniature Y-mazes to measure spontaneous alternation behavior by scoring the number of three consecutive arm entries over a period of ten minutes [see Materials and Methods, Fig. 3A, 83]. These experiments showed that although TDP-43 OE results in increased overall movement (males: *TDP-43*^*WT*^*::YFP* in an *Oregon-R background*, 146.00 $\pm$ 7.51 mm vs. *Oregon-R*, 106.19 $\pm$ 8.18 mm, *P* = 0.018; females: *TDP-43*^*WT*^*::YFP* in an *Oregon-R background*, 110.53 $\pm$ 7.51 mm vs. *Oregon-R*, 81.12 $\pm$ 5.24 mm; Fig. 3b; for means of individual replicates see Additional file 2: Table S3), percent alternation is significantly decreased in young adult males (*TDP-43*^*WT*^*::YFP* in an *Oregon-R background*, 0.57 $\pm$ 0.01 vs. *Oregon-R*, 0.61 $\pm$ 0.01, *P* = 0.012) and females (*TDP-43*^*WT*^*::YFP* in an *Oregon-R background*, 0.60 $\pm$ 0.02 vs. *Oregon-R*, 0.65 $\pm$ 0.01, *P* = 0.006; Fig. 3c), consistent with TDP-43 proteinopathy causing working memory deficits in the MB circuit.

### TDP-43 expression in the MB circuit causes sleep alterations

**Fig. 4:**
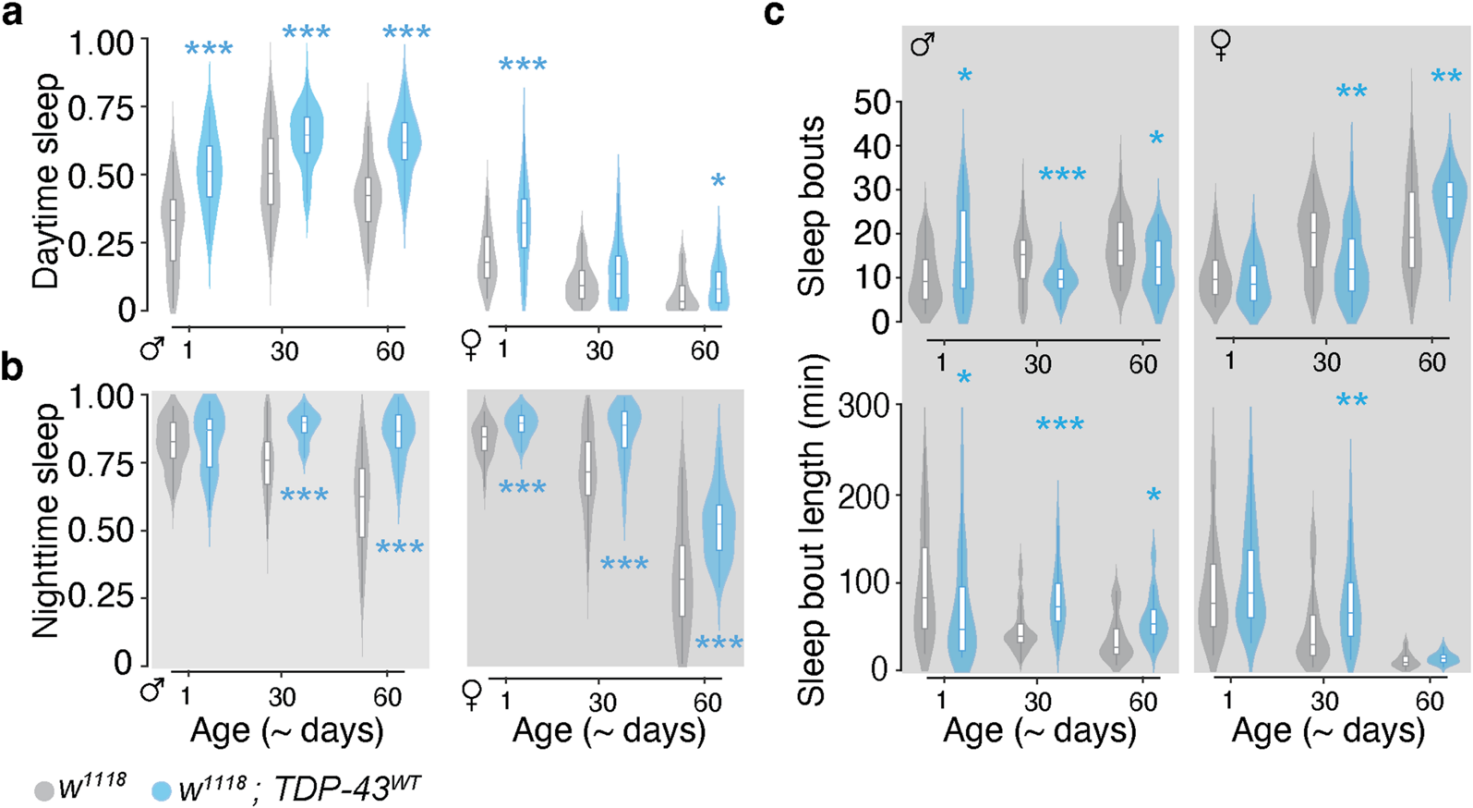
TDP-43^WT^ overexpression in MBNs reduces arousal, increasing day and night sleep. **a** Proportion of time flies spent sleeping during the day and **b** at night. **c** Sleep fragmentation assessed by number of sleep bouts (top panel) and mean bout length (bottom panel) during the night. Male data on the left and female data on the right in each panel. Data for TDP-43^G298S^ presented in Fig. 4-supplement 1. * = *P* < 0.05; ** = *P* < 0.01; *** = *P* < 0.001

In humans, frontal and temporal lobe degeneration is associated with increased daytime somnolence [103], but overall sleep disturbances are highly variable in FTD and may be detectable in earlier stages than in AD [104]. To determine whether TDP-43 proteinopathy in the MB circuit alters sleep, we used *Drosophila* Activity Monitors (DAMs, see Materials and Methods) to measure total daytime and nighttime sleep. Our experiments show that on average, both male and female TDP-43 OE flies appear to be lethargic; they spent a greater proportion of their time sleeping during both the day and night compared to controls (approximately 11% more for TDP-43^WT^ OE and approximately 8% more for TDP-43^G298S^ OE). However, there are subtle differences in how TDP-43 OE affects sleep patterns based on age and sex. While males consistently sleep more during the day, this effect is weaker in females where we found trends towards increased daytime sleep at some but not all age time points in each genotype (Fig. 4a; Additional file 2: Table S4a). At night, males and females also exhibited a general tendency towards increased sleep with the exception of young TDP-43 OE adult males which, when compared to *w*^*1118*^ controls, showed signs of sleep fragmentation as evidenced by more frequent, shorter sleep bouts (Figs. 4b, c for TDP-43^WT^ OE and Additional file 1: Fig. S1-supplement 1b, c for TDP-43^G298S^ OE*;* Additional file 2: Table S4c sleep bout length and S4c sleep bout number).

Interestingly, both humans and flies show decreased nighttime sleep with age [105]; in line with this, *w*^*1118*^ flies of both sexes showed age-related declines in nighttime sleep, whereas TDP-43 OE males showed no such declines (all pairwise comparisons *P* > 0.05) and female declines appear less steep (TDP-43^WT^ OE, Fig. 4b; TDP-43^G298S^ OE, Additional file 1: Fig. S4-supplement 1; Additional file 2: Table S4a), consistent with our findings that TDP-43 proteinopathy causes lethargy. By middle age, TDP-43^WT^ OE males showed a pattern of longer, less frequent nighttime sleep bouts as compared with *w*^*1118*^ (Fig. 4c). Within each genotype, female w^1118^ and TDP-43^WT^ OE flies exhibited age-related sleep fragmentation as evidenced by a greater number of shorter bouts at 60–63 days compared with 1–3 days. TDP-43 OE flies still spent a greater proportion of their time sleeping than controls (Fig. 4b; Additional file 2: Table S4a), an effect driven by longer sleep bouts at this aged time point (Fig. 4c; Additional file 2: Table S4c). Taken together, these results indicate that TDP-43 OE in the MB circuit causes sleep deficits resembling those reported in FTD patients, including increased sleep during the day and sleep fragmentation at night, albeit the latter was detected in young males only. Notably, the sleep fragmentation phenotype occurs concomitant with the nuclear depletion of TDP-43 and before axonal degeneration suggesting that this may be a direct consequence of TDP-43 mislocalization while lethargy is likely an indirect consequence of large-scale neuronal degeneration.

### TDP-43 overexpression in Kenyon cells is sufficient to reduce lifespan in Drosophila

**Fig. 5:**
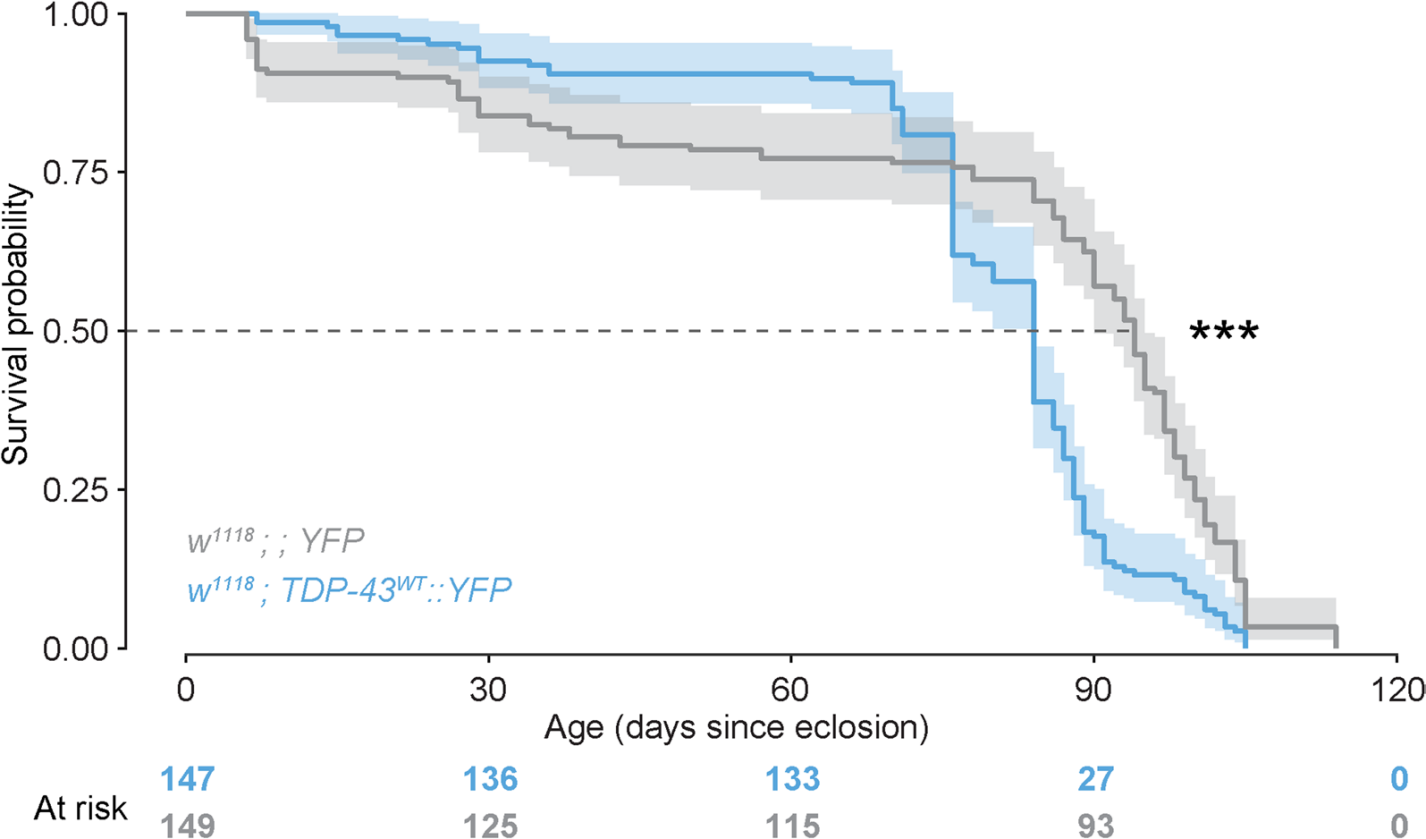
TDP-43 overexpression in MBs is sufficient to reduce lifespan. Data from male and female flies pooled for analysis. Number of flies N = 149 for YFP controls and 147 for TDP-43^WT^ YFP expressing flies). Data for TDP-43^G298S^ presented in Fig. 5-supplement 1. *** *P* < 0.001

Life expectancy for FTD patients after diagnosis is approximately 8–12 years and, while patients do not often die from the disease itself, there is a measurable, if subtle, effect on life expectancy [106, 107]. We therefore tested whether TDP-43 proteinopathy affects lifespan in our fly model and found that overexpression of TDP-43 in MBNs significantly reduces median survival time (TDP-43^W^ OE, 84 days, 95% CI [38, 38]; TDP-43^G298S^ OE, 89 days, 95% CI [27, 108]) when compared with *YFP* controls (94 days, 95% CI [107, 109]; *P* < 0.0001 and *P* = 0.028, respectively; Fig. 5 and Additional file 1: Fig. S5-supplement 1). These results indicate that overexpression of TDP-43 in a subset of MBNs in flies is sufficient to produce a nearly 10% reduction in median lifespan.

### mRNAs enriched with TDP-43 in mushroom body neurons show partial overlap with those previosuly identified in motor neurons and include components of the Wnt/Wg signaling pathway

To understand how TDP-43 overexpression influences the MBN proteome, we immunoprecipitated YFP-tagged TDP-43^WT^ or TDP-43^G298S^ from *Drosophila* heads overexpressing TDP-43 YFP in MBNs using the *SS01276* split GAL4 driver. Next, we used DESeq2 [91] to assess differential expression of mRNAs enriched in TDP-43 complexes compared to the whole brain transcriptome. We chose to identify TDP-43-enriched candidate mRNA targets using young adult flies (1–3 days post-eclosion), before age-dependent axonal neurodegeneration is detected in the MB lobes, to avoid detection of gene expression alterations that could be an indirect consequence of neurodegeneration. Of the 13,302 transcripts detected in the fly brain, we identified 1055 and 1393 candidate mRNA targets based on their enrichment in TDP-43^WT^ YFP and TDP-43^G298S^ YFP complexes, respectively (Log2FC > 1 compared to the brain transcriptome, *P*_adj_ < 0.05) (Fig. 1a, 6a). A total of 876 candidate mRNAs were shared by both variants (83% of targets in TDP-43^WT^ OE and 63% of targets in TDP-43^G298S^ OE), for a total of 1572 mRNAs enriched across both models (with 179 unique to TDP-43^WT^ OE and 517 to TDP-43^G298S^ OE, respectively). Notably, differential expression analyses also recovered previously identified TDP-43 target mRNAs including *futsch* (TDP-43^WT^ OE Log2FC = 1.81, *P*_adj_ = 2.66E−07; TDP-43^G298S^ OE Log2FC = 2.14, *P*_adj_ = 9.57E−10) [56] and *dscam2* (TDP-43^WT^ OE Log2FC = 1.29, *P*_adj_ = 6.01E−04; TDP-43^G298S^ OE Log2FC = 1.81, *P*_adj_ = 2.42E−3) [110].

**Fig. 6:**
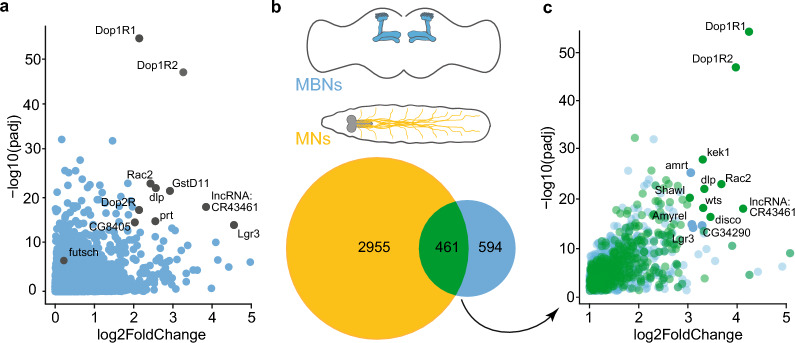
mRNAs enriched with TDP-43 in *Drosophila* models of proteinopathy in FTD and ALS. **a** Volcano plot displaying mRNAs enriched with TDP-43. Y-axis depicts Log2 Fold Change after subtraction of YFP control values (see Materials and Methods). A small number of targets showing greatest enrichment with high confidence are highlighted with blue circles (log2 Fold Change > 2 and *P* < 1 × 10^–14^). **b** Schematic showing subsets of neurons in fly models of FTD (MBs, above) and ALS (MNs, below), and Venn diagram depicting enriched mRNAs unique to MB and MN circuits, and overlapping between models. **c** Volcano plot displaying mRNAs enriched in TDP-43 complexes that are MB-specific (blue) or shared between MB and MN models (green); saturated green circles indicate shared targets that show log2 Fold Change > 3 and *P* < 1 × 10^–14^; blue circles indicate MB targets with log2 Fold Change > 2 and *P* < 1 × 10^–14^. Data for TDP-43^G298S^ presented in Fig. 6-supplement 1

While TDP-43 has been found to regulate mRNA processing in a cell type specific manner, there is also overlap among TDP-43 splicing targets in neuronal and muscle cell lines [111]. Consistent with these findings, we found that nearly 44% of TDP-43^WT^ OE and 42% of TDP-43^G298S^ OE highly associated mRNAs in MBNs overlapped with those we identified previously in motor neurons (MNs) when modeling ALS by overexpressing TDP-43 with the D42 GAL4 driver (Fig. 6b, Additional file 1: Fig. S6-supplement 1c).

Functional annotation of the mRNA candidate targets in the MBs identified “neuron-specific” as well as “neuromuscular junction” molecular pathways in both the TDP-43^WT^ OE and TDP-43^G298S^ OE models (Additional file 1: Fig. S6-supplement 2a). Components of the Wingless (Wg/Wnt) signaling pathway including the *frizzled* (*fz*) and *frizzled 2* (*fz2*) [112] receptors, and *dally-like protein* (*dlp*), a heparan sulfate proteoglycan (HSPG) that interacts with multiple Wnt ligands [113] were overrepresented in these analyses (Additional file 1: Fig. S6-supplement 2B). Interestingly, *dlp* mRNA was also found to be enriched with TDP-43 in fly MNs, where TDP-43 proteinopathy causes depletion of Dlp from the neuromuscular junction and simultaneous accumulation of Dlp puncta in cell bodies [38]. To validate that *dlp* mRNA is a target of TDP-43 in MBNs as predicted by RNA seq, we used RNA IPs followed by RT-qPCR and confirmed that *dlp* mRNA is enriched in immunoprecipitated TDP-43^WT^ and TDP-43^G298S^ complexes compared to input (Additional file 1: Fig. S6-supplement 1).

To gain insights into circuit specific aspects of TDP-43 proteinopathy, we also analyzed the pool of mRNAs significantly enriched with TDP-43 in MBNs only and not shared with MNs. For TDP-43^WT^ OE we found 461 enriched mRNAs that are shared in both MB and MN, and 594 mRNAs uniquely enriched in MBs (Fig. 6). DAVID analyses of the unique candidate targets for TDP-43^WT^ OE in MBs only uncovered several kegg pathways including “Basal transcription factors”, “RNA polymerase”, “Ribosome biogenesis” and “Spliceosome” (Additional file 2: Table S6A). For TDP-43^G298S^ we found 592 enriched mRNAs that are shared in both MB and MN, and 802 mRNAs uniquely enriched in MBs (Additional file 1: Fig. S6-supplement 1). DAVID analyses of TDP-43^G298S^ specific targets in the MB circuit revealed several significantly represented pathways including “mTOR signaling pathway”, “Other types of O-glycan biosynthesis”, “TGF-beta signaling pathway”, “Nicotinate and nicotinamide signaling pathway”, “Autophagy”, “Nucleotide metabolism” and “Spliceosome” (Additional file 2: Table S6B).

Taken together, these results identify shared candidate targets among MBNs and MNs including the Wg/Wnt signaling pathway and highlight novel, MB specific candidate mRNA targets of TDP-43 proteinopathy across ALS/FTD relevant neuronal circuits.

### TDP-43 proteinopathy causes severe age-related loss of Dally-like protein in MBNs

**Fig. 7:**
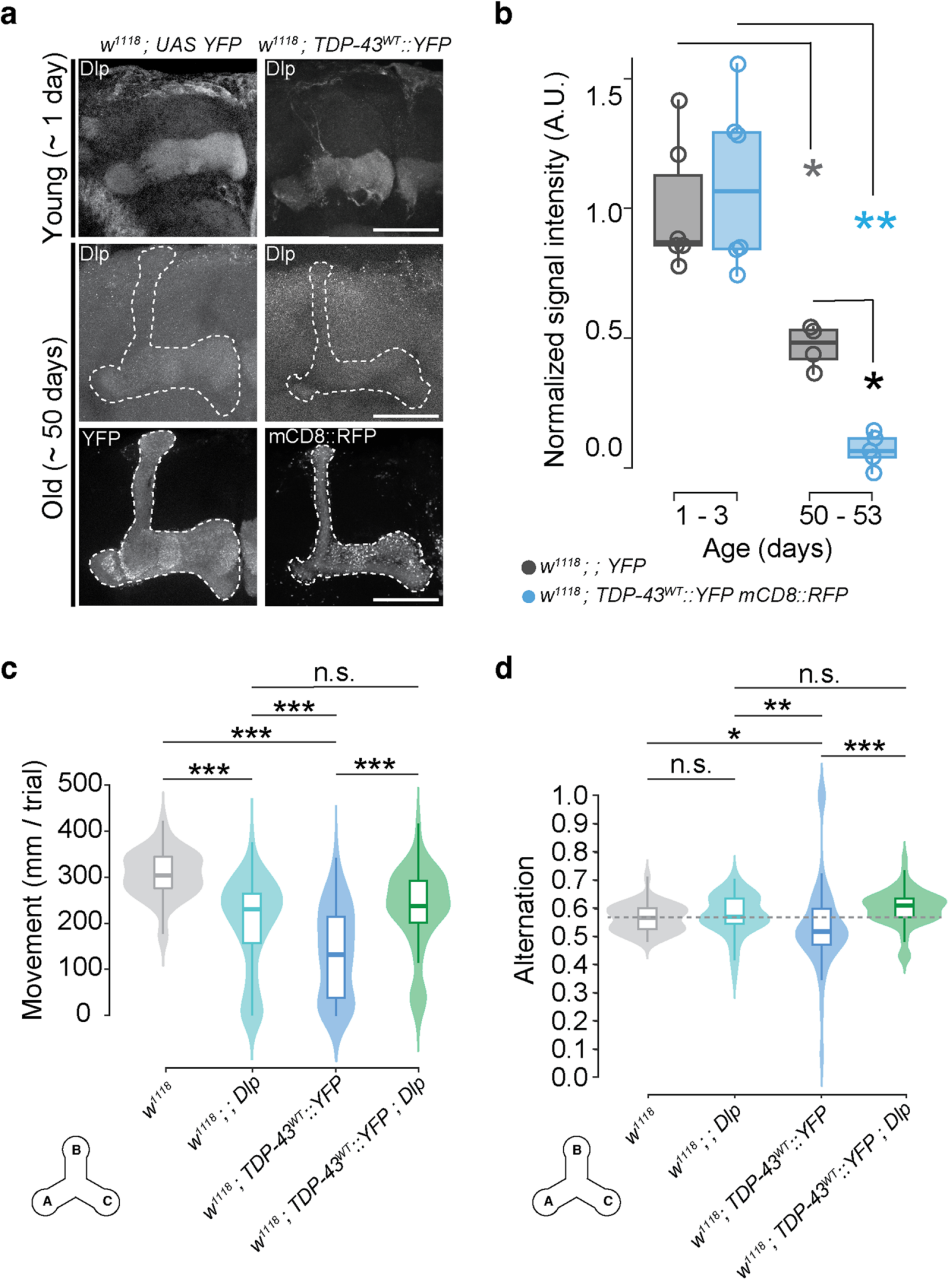
Dally-like protein is a target of TDP-43^WT^ in MBNs where it mediates TDP-43 dependent working memory deficits. **a** Dlp antibody labelling in mushroom bodies shows age and TDP-43 dependent reduction. Genotypes (*w*^*1118*^*; YFP* and *w*^*1118*^*; TDP-43*^*WT*^*::YFP mCD8::RFP*), stainings (Dlp, YFP, RFP) and ages (1 and ~ 50 days old), as indicated. **b** Quantification of age and TDP-43 dependent changes in Dlp signal intensity within MBLs. **c** Mean distance moved and **d** alternation per trial in males overexpressing Dlp (*w*^*1118*^*;; dlp OE),* TDP-43^WT^ (*w*^*1118*^*; TDP-43*^*WT*^*::YFP)* or both *(w*^*1118*^*; TDP-43*^*WT*^*::YFP; dlp OE*) compared to *w*^*1118*^ genetic background controls. Grey dotted line represents control group median. Scale bar = 50 mm. Data for TDP-43^G298S^ presented in Fig. 7-supplement 1. * = *P* < 0.05, ** = *P* < 0.01

Given *dlp* mRNA’s enrichment with TDP-43, we hypothesized that the expression of Dlp protein, a Wg/Wnt regulator, may be affected in MBNs, as we previously found in MNs [38]. While Dlp has been shown to be highly expressed in developing MBN axons, where it is thought to be involved in axon guidance [114], its expression and function in adult MBNs has not been characterized. We found that, despite *dlp* mRNA being enriched in TDP-43 complexes in young adult brains, the levels and distribution of Dlp protein in MB axons of TDP-43 OE flies are comparable with controls at that age (Figs. 7a, b). Interestingly, Dlp levels in MB axons decline with age in both controls and TDP-43 OE flies, however while Dlp is still visible in control fly brains at 50 days, Dlp is nearly undetectable in the MB axons of TDP-43 OE flies (Figs. 7a, b, P < 0.05). A similar age dependent reduction in Dlp expression was also detected in the context of TDP-43^G298S^ OE in MBNs (Additional file 1: Fig. S7-supplement 1). These findings are consistent with *dlp* mRNA being a target of TDP-43 in MBNs and suggest mRNA localization and/or translation as possible mechanisms for the observed reduction in Dlp protein expression.

### Dlp over expression rescues TDP-43 dependent working memory deficits

We next evaluated the physiological significance of *dlp* mRNA as a target of TDP-43 in MBs. To address this, we tested whether overexpression of *dlp* mRNA (dlp OE) in the context of TDP-43 proteinopathy could mitigate TDP-43 dependent working memory deficits. Using the Y-maze working memory assay we found that the percent alternation deficits displayed by TDP-43^WT^ OE flies could indeed be mitigated by dlp OE in MBNs (Fig. 7d). To account for altered movement caused by TDP-43 OE (*TDP-43*^*WT*^*::YFP* $,$ 147.52 $\pm$ 13.52 mm, *w*^*1118*^ $,$ 229.89 $\pm$ 7.43 mm, P < 0.0001, see Fig. 7c), we also compared distance-scaled alternations across genotypes and found that TDP-43 dependent alternation deficits were still mitigated by dlp OE* (TDP-43*^*WT*^*::YFP*, 0.0221 $\pm$ 0.0028 alternation/mm, *TDP-43*^*WT*^*::YFP dlp OE*, 0.0286 $\pm$ 0.0036 alternation/mm, P < 0.0001) while no significant difference were detected between co-overexpression of TDP-43 and Dlp, and dlp OE alone (*TDP-43*^*WT*^*::YFP dlp OE* $,$ 0.0286 $\pm$ 0.0036 alternation/mm, *dlp OE* $,$ 0.0252 $\pm$ 0.0032 alternation/mm, P > 0.05, not shown). We note that although total movement in the Y-maze assay is influenced by the genetic background (*w*^*1118*^ versus *OR-R*, compare Figs. 3b and 7c), the significant reduction in spontaneous alternation is consistent for TDP-43 OE flies in both cases (Figs. 3c and 7d). These experiments show that *dlp* OE in the MB circuit rescues the reduced alternation behavior caused by TDP-43^WT^ OE in the Y-maze assay and suggest that Dlp dysregulation mediates TDP-43 dependent working memory deficits in MBNs. Notably, this rescue effect is not due to reduced TDP-43 expression or to diluted GAL4 activity by the presence of an additional UAS promoter, as evidenced by similar TDP-43 levels with or without dlp OE (Additional file 1: Fig. S7-supplement 2, supplement 3). Surprisingly, OE of TDP-43^G298S^ in MBNs did not cause a significant spontaneous alternation phenotype (see raw data in Additional file 2: Table S7). Taken together, these findings support the notion that *dlp* mRNA is a functional target of TDP-43 in vivo and mediates, at least in part, TDP-43 dependent, dementia relevant phenotypes in *Drosophila*.

### Several mRNA candidate targets of TDP-43 in MBNs including GPC6, a human ortholog of dlp, exhibit altered expression in FTD patient brains

To validate our findings from *Drosophila* models in patients, we compared the list of mRNAs enriched with TDP-43 in fly MBNs with existing, whole transcriptome, bulk RNA seq data sets [115] and found several orthologs of human genes exhibiting altered expression in the frontal cortices of FTD patients. These include the Ras family member RHOQ (*Rac2*, *TDP-43*^*WT*^ Log2FC = 3.68, Padj = 9.66E−24), the glypican GPC4 (*dlp*, *TDP-43*^*WT*^ Log2FC = 3.33, Padj = 9.14E−23), heat shock 70 kDa protein 2, HSPA2 (*Hsp70Bb*, *TDP-43*^*WT*^ Log2FC = 2.77, Padj = 1.59E−7), and the frizzled family receptor protein FZD7 (*fz*, *TDP-43*^*WT*^ Log2FC = 2.18. Padj = 4.25E−12).

**Fig. 8:**
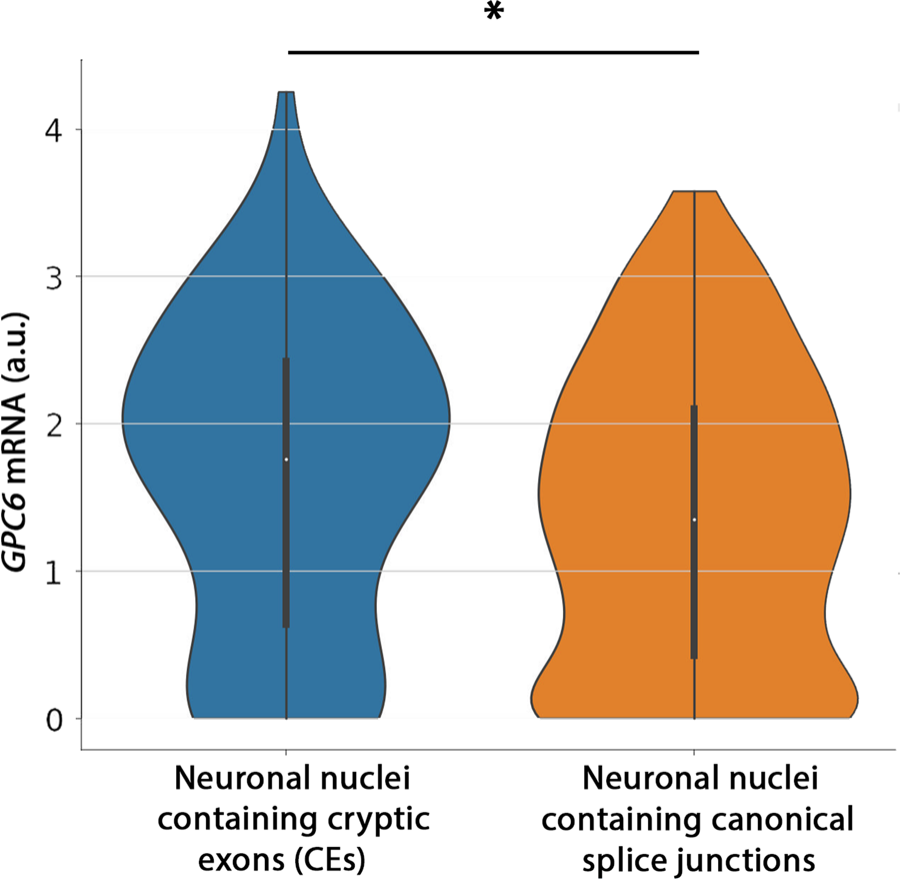
*GPC6* mRNA is significantly increased in neuronal nuclei containing TDP-43 associated cryptic exons compared to normal nuclei within FTD patient brains. *GPC6* mRNA levels as shown, in nuclei in which TDP-43 associated cryptic exons (*e.g., STMN2*, *KALRN*) have been identified (N = 87 neurons) compared to nuclei containing canonical splice junctions (N = 377 neurons). *P* = 0.015. *a.u.* arbitrary units

In addition to these bulk RNA seq data sets, we also utilized a recent, single nuclei RNA seq (snRNAseq) study of FTD patients with TDP-43 pathology and harboring mutations in C9orf72, the most common form of inherited ALS/FTD [94]. When comparing our mRNA candidate targets identified in *Drosophila* with transcripts altered in single neuronal nuclei containing TDP-43 associated cryptic exons (*e.g.*, *STMN2*, *KALRN*, N = 87) versus neurons in which canonical splice junctions were present (N = 377), we found several human orthologs to be altered including *dlp*’s ortholog, *GPC6* mRNA, which was found to be significantly upregulated (log normalized mean expression of 1.65 in cryptic exon containing nuclei, 1.35 in nuclei containing canonical splice junctions, *P* = 0.015, Fig. 8, Table 1 and Additional file 2: Table S8). Interestingly, several candidate mRNA targets of TDP-43, both shared between MBNs and MNs, or unique to MBNs in flies have human orthologs that are differentially expressed in neuronal nuclei containing TDP-43 associated cryptic exons in FTD patient brains (Tables 1 and 2). These data suggest that nuclear depletion of TDP-43, as evidenced by the presence of TDP-43 associated cryptic exons, causes gene expression alterations in several TDP-43 candidate mRNA targets including *GPC6*, linking this candidate target identified and functionally validated in *Drosophila* to TDP-43 pathology in FTD patient brains.

**Table 1:** Shared TDP-43 candidate mRNA targets, between mushroom body and motor neurons, identified in *Drosophila* and found to be transcriptionally altered in single neuronal nuclei from FTD patient brains

Human gene	Fly gene	HGNC ID	DIOPT score	Log2FC	FDR	Gene function	Association with neurodegenerative disease
GPC6	dlp	HGNC:4454	11	0.411254	0.000270	GPI anchored heparan sulfate proteoglycan	AD risk factor in two GWAS studies [116, 117]
ENC1	CG9426	HGNC:3345	4	0.354832	0.000002	Actin cytoskeleton associated	Candidate risk factor in progression from asymptomatic to symptomatic AD [108]
FRAS1	cv-2	HGNC:19185	3	0.350035	0.000000	Binds BMP receptor and HSPG	Component of extracellular matrix altered in AD brains [118]
GRIA4	Ir68a	HGNC:4574	3	0.319357	0.000075	Glutamate ionotropic receptor AMPA type subunit 1	De novo variants associated with ID [119]; upregulated in CSF fluid of ALS patients and negatively correlated with severity [120]
OPCML	DIP-alpha	HGNC:8143	8	− 0.307174	0.000000	Synaptic cell adhesion molecule	This class of proteins associated with AD [121]; LSAMP was identified in this paper as well
LSAMP	DIP-alpha	HGNC:6705	8	− 0.327900	0.000089	Adhesion molecule involved in neuronal growth and axonal targeting	Altered expression in FTD and AD [122, 121]
KCNH5	eag	HGNC:6254	12	− 0.329006	0.000181	Potassium channel	Mutations assocaited with seizure disorders [123]; Upregulated in chronic acive lesion of Multiple Sclerosis [124]
GABRG3	Rdl	HGNC:4088	3	− 0.475203	0.000000	GABA gated chloride channel	Downregulated in AD mouse model [125]; Linked to neurodegeneration via role as transcriptional target of the retinoic acid receptor RARβ [126]

DIOPT score—ortholog identifier tool [95]. Log2FC and FDR—differential expression data for human orthologs, identified in neuronal nuclei from FTD patient brains exhibiting the molecular signature of TDP-43 pathology [see Materials and Methods, and 94. Gene function and association with neurodegenerative disease, as shown

**Table 2:** Candidate mRNA targets of TDP-43 in mushroom body neurons, identified in *Drosophila* and found to be transcriptionally altered in single neuronal nuclei from FTD patient brains

Human gene	Fly gene	HGNC ID	DIOPT score	Log2FC	FDR	Gene function	Association with neurodegenerative disease
NFIB	NfI	HGNC:7785	11	0.374332	0.005697	DNA-binding transcription factor activity	Altered expression in AD [127]
PLPPR4	wun	HGNC:23496	3	0.370091	0.000000	Lipid phosphate phosphatase	Altered expression in AD patient cortices [128]
ENC1	dbo	HGNC:3345	4	0.354832	0.000447	Substrate-specific adapter of an E3 ubiquitin-protein ligase complex	Altered expression in AD [108]
TMTC1	Tmtc4	HGNC:24099	3	0.338313	0.006021	Transmembrane O-mannosyltransferase targeting cadherins 4	Altered expression in mouse models of AD [129]
GRIA4	GluRIIE	HGNC:4574	7	0.319357	0.008298	Glutamate receptor	Protein levels reduced across different dementias [130]; altered expression in AD [127]
NCALD	CG5890	HGNC:7655	3	− 0.421750	0.000044	Neuronal calcium sensor	Decreased in AD [131]
FOXP2	FoxP	HGNC:13875	8	− 0.434982	0.002966	Forkhead box transcription factor	Linked to speech and language disorders; downstream targets include motor proteins [132]

DIOPT score—ortholog identifier tool [95]. Log2FC and FDR—differential expression data for human orthologs, identified in neuronal nuclei from FTD patient brains exhibiting the molecular signature of TDP-43 pathology [see Materials and Methods, and 56]. Gene function and association with neurodegenerative disease, as shown. Note: ENC1 and GRIA4 are present on both MB-MN shared and MB unique lists due to homology to distinct genes identified in *Drosophila*

## Discussion

Neurodegenerative diseases have historically been classified by shared symptomology and post-mortem pathology. More recently, the integration of molecular markers and genetic testing has uncovered evidence that cell-type susceptibility and dysregulation of specific molecular pathways can interact to produce similar clinical presentations across different disease etiologies [133]. These findings highlight convergent pathomechanisms during the degeneration process [3, 22] and suggest inroads for better understanding of these complex diseases.

A common pathological feature of several neurodegenerative diseases including ALS, FTD, and other dementias is the nuclear depletion of the RNA binding protein TDP-43 accompanied by the accumulation of cytoplasmic, ubiquitinated foci [13–16, 18, 134]. Since dementia is often confirmed post-mortem by the presence of TDP-43 pathology, animal models play an important role in elucidating the molecular mechanisms underlying TDP-43 proteinopathy and its role in neurodegeneration. Among these, fly models have proven their utility in this research landscape in part because the availability of powerful molecular and genetic tools allowing multiple hypotheses to be pursued in parallel on a large scale. Indeed, fly models of TDP-43 proteinopathy based on loss of function or overexpression in the entire nervous system [55, 135, 136], or in subsets of neurons including associative regions of the central brain [54], motoneurons [43] or the retina [137] have revealed a broad repertoire of altered pathways that have subsequently been confirmed in patient tissues [37, 38, 56].

It has been previously shown that overexpression of TDP-43 in all MBNs using the driver line OK107 GAL4 (RRID:BDSC_854) caused age-dependent cell loss and axonal degeneration in MBs, however degeneration severity varied widely across nearly isogenic individuals [54]. This variability could be in part attributed to driver line “leakiness” as OK107 GAL4 drives expression beyond MBs, in optic lobe neuropils [138, 139] and neurosecretory cells [140, 141]. To develop a robust, MB specific model of TDP-43 driven dementia in *Drosophila melanogaster*, we leveraged a split GAL4 driver line with limited expression to a subset of Kenyon cells (γ, α/β MBNs). Notably, this model exhibits nuclear depletion and cytoplasmic accumulation of TDP-43, accompanied by age-dependent axonal degeneration and cell loss, all of which recapitulate key aspects of disease pathology. Importantly, the onset of FTD relevant behavioral symptoms is detectable prior to widespread degeneration, as observed in human disease. Additionally, this model identifies both novel, MB specific, and motor neuron-shared mRNA candidate targets that have previously been associated with TDP-43 pathology. Of the latter, here we chose to focus on the glypican Dlp, a Wg/Wnt signaling regulator. We show that Dlp is a functional target of TDP-43 in the MB circuit that mediates, in part, TDP-43 dependent working memory deficits in the fly model.

Neurodegeneration in ALS/FTD is thought to be driven by both loss of nuclear function, as some nuclei become devoid of TDP-43, and cytoplasmic gain of toxic function, as evidenced by TDP-43 accumulation in cytoplasmic puncta. While thus far invertebrate models of TDP-43 proteinopathy have not faithfully recapitulated TDP-43 mislocalization [e.g., 98, 142], here we found that TDP-43 over-expression in MBNs more closely resembles disease pathology. Indeed, TDP-43 displays normal localization in the juvenile stage but becomes depleted from the nucleus and mislocalized to the cytoplasm in young adults. Although changes in TDP-43 localization could reflect developmental regulation [143], our findings that the number of cells with nuclear TDP-43 depletion is reduced in young (1–3 days old) compared to old (50–53 days old) adult flies, concommitant with an overall reduction in MB neuron numbers, suggests that TDP-43’s shift in distribution from the nucleus to the cytoplasm is toxic.

Axonal degeneration is evident only in older adult flies and differentially affects MBNs that form the α/β and γ lobes, with the latter showing the greatest degeneration severity. This is particularly interesting because MBNs that form the γ lobe are embryonic in origin and therefore older than the pupal-born MBNs that form the α/β lobes [99, 144, 145], highlighting the aging component of neurodegeneration. It is also possible that the differences in the onset and effects of TDP-43 proteinopathy we observed in γ lobe MBNs may be due to their remodeling during metamorphosis [99], which may increase their vulnerability to TDP-43. Taken together, these findings parallel differential susceptibility observed in human cortical neurons [146, 147]. Given the availability of split GAL4 driver lines for subsets of MBNs [97] future studies could focus on better understanding of what drives this susceptibility by modeling TDP-43 proteinopathy in various neuronal subpopulations within the MB circuit. It will be interesting to combine these powerful genetic tools with single cell RNA seq efforts in patients and flies in order to pinpoint specific cell sub-types that may be more vulnerable.

Frontotemporal lobar degeneration with TDP-43 proteinopathy (FTLD-TDP) is pathologically classified based on the cortical layers affected, the number of dystrophic neurite, and the extent of neuronal cytoplasmic inclusions [21]. This pathology is also commonly observed in Alzheimer’s disease and dementia with Lewy bodies cases that exhibit TDP-43 proteinopathy, although different brain regions are selectively affected [14, 19]. Interestingly, we find that neuronal cytoplasmic inclusions are absent in juveniles, appear first in young adult brains and increase in size during aging. This neuropathology precedes observations of dystrophic neurites, which are first visible around middle age, increase in severity during aging, and ultimately degenerate. This age-dependent pathology observed in the fly brain parallels that observed in human disease [148] and provides a platform for studying the progression of neurodegeneration in the genetically tractable *Drosophila* model.

In humans, behavioral symptoms of FTD precede widespread degeneration by years suggesting that behavioral deficits arise prior to neuronal pathology, in the absence of complete loss of nuclear TDP-43 function or cytoplasmic accumulation [149]. Therefore, we chose to focus primarily on working memory and sleep deficits using young adult flies, in which a subset of MBNs show nuclear depletion accompanied by widespread TDP-43 mislocalization to the axonal cytoplasm while MBN axons still appear largely intact. At this young age, we found both working memory and sleep fragmentation phenotypes, albeit the latter were only significant in males. We also assessed these behaviors in aged flies, however the distinct age-dependent deficits exhibited by the controls themselves confounded the detection of TDP-43 specific phenotypes.

Behavioral variant frontotemporal dementia (bvFTD) is diagnosed based on often subtle behavioral symptoms including a lack of empathy, increased apathy, dysinhibition and deficits in executive function [150]. FTD was classically distinguished from AD using a perceived lack of memory deficits, however recent patient studies and meta-analyses indicate that memory deficits are common in bvFTD and at times indistinguishable between AD and bvFTD [133, 151], challenging the validity of the exclusion of episodic memory deficits in the clinical diagnoses of bvFTD [152]. Although sleep symptoms are notoriously variable and understudied across different FTD diagnoses, the most common sleep disturbances observed in bvFTD patients are sleep fragmentation and daytime sleepiness that may or may not be accompanied by insomnia [103, 104, 153]. Our findings of limited sleep fragmentation and daytime sleepiness highlight potential differences between the fly model and human disease presentation. That said, the limited yet specific deficits caused by TDP-43 OE in MBNs provide a robust measurable sleep disruption that can serve as an organism-level output for future molecular or pharmacological intervention experiments.

In addition to observed behavioral deficits, flies with TDP-43 proteinopathy show an approximately 10% reduction in median lifespan. This may be roughly comparable to human patients where FTD exerts a subtle effect on lifespan. In humans, mean age at diagnosis is 61.9 for early onset disease (< 65 years) with mean survival after diagnosis around 8 years [106]. Although the effect on survival could be caused by socioeconomic status and access to care, the lifespan of FTD patients is reduced in comparison with the general population in most countries were clinical data are collected [e.g., 78.8 years in the United States 154]. Effects on lifespan may be far more severe than a simple comparison of life expectancy would indicate, as Loi et al. [107] report an increase in mortality risk for FTD patients when compared with age-matched controls. From the perspective of a fly model, it is somewhat surprising to see a lifespan effect given that MBNs comprise a small fraction of total neurons in the fly brain and are often thought to be largely dispensable for viability [63]. There is evidence that lifespan is regulated at least in part by the α/β MBNs [109], a subset of MBNs included in our model, however loss of key MB proteins such as DCO [155] or over-expression of vertebrate TAU in MBNs result in severe memory deficits without affecting lifespan [156]. Although lifespan reduction could be due to secondary effects such as food consumption or other factors, our model reproduces an important, subtle characteristic of FTD and provides additional evidence that specific populations of MBNs may play a role in lifespan regulation in flies.

Sequestration of mRNAs is one the mechanisms by which TDP-43 is thought to contribute to neurodegeneration and identifying candidate mRNA targets has helped better understand disease etiology [39, 157]. Although our model is based on TDP-43 overexpression, we note that among the mRNAs enriched with TDP-43 in MBNs we found 12 of 15 physiological targets of TDP-43 previously identified bioinformatically as potential targets of *Drosophila* TDP-43 (*i.e.*, TBPH) based on UG-richness [158]. In future studies, it will be interesting to explore the relationship between splicing and translational targets of TDP-43. Furthermore, the overlap and specificity of some targets for MNs versus MBNs may provide insights into shared mechanisms and neuronal vulnerability across neurodegenerative diseases exhibiting TDP-43 proteinopathy.

Functional analyses of the mRNAs associated with TDP-43 identify numerous cellular pathways including Wg and Hippo that have been previously implicated in sleep regulation in flies [159]. Additionally, our findings of dopamine receptors as candidate mRNA targets is consistent with findings that loss of mesocortical dopaminergic tracts and dopamine receptors in the frontal lobes could contribute to the behavioral symptoms in FTLD [160].

For functional validation we chose to focus on *dlp*, an mRNA target of TDP-43 that we previously identified in the ALS model of TDP-43 proteinopathy, and a known regulator of Wg signaling. Indeed, Wg signaling is dysregulated in ALS [161, 162], mRNAs associated with Wg/Wnt pathway are enriched in FTD patient frontal cortices [115] and WNT1 and Granulin (GRN), an FTD linked gene, have been shown to regulate each others’ expression in human neuronal progenitors [163]. Despite these connections, the mechanisms by which the Wg/Wnt pathway is implicated in TDP-43 based pathophysiology in FTD or other dementias remains poorly understood. Using genetic interactions, we found that *dlp* OE in MBNs mitigates TDP-43 dependent working memory deficits, as evidenced by improved alternation in the Y maze assay. Taken together, these results support the notion that Wg/Wnt signaling is altered in TDP-43 associated neurodegeneration and modulating its activity via Dlp mitigates behavioral deficits caused by TDP-43 proteinopathy. Further substantiating these findings, we found that the expression of *GPC6* mRNA, a human ortholog of *dlp,* is altered in FTD patient brains [94], specifically in neuronal nuclei that exhibit the molecular signature of TDP-43 depletion (*i.e.*, cryptic exon inclusion in specific TDP-43 transcriptional targets), consistent with a link between Dlp/GPC6 and TDP-43 pathology. We speculate that this increase in *GPC6* mRNA in neurons with TDP-43 nuclear depletion may reflect a compensatory upregulation caused by a cytoplasmic decrease in protein levels that we detected in *Drosophila* MBN axons. Indeed, the same study by Gittings, Alsop et al. [94], identifies an increase in the expression of genes related to oxidative phosphorylation, ATP synthesis and energy metabolism in the cryptic exons containing cells isolated from FTD patients, suggesting a compensatory mechanism to counteract documented reductions in ATP synthesis and overall mitochondrial function [reviewed in 164]. Although it is also possible that Dlp/GPC6 are differently regulated in flies and humans, the link to TDP-43 proteinopathy is preserved, highlighting the ability of the *Drosophila* models to identify functionally relevant disease targets. Interestingly, recent GWAS studies identified *GPC6* as a risk factor for AD in African Americans [116, 117]. In future studies it will be interesting to see how different mRNA targets, beyond *dlp/GPC6* mitigate specific phenotypic aspects of FTD and identify additional targets across the spectrum of TDP-43 proteinopathies.

### Supplementary Information

**Additional file 1:** **Figure 1-supplement 1.** Maximum intensity projections showing split Gal4 driver line *SS01276* expression in the 3rd instar larva. **Figure 1-supplement 2.** TDP-43^G298S^ localization in MBN cell bodies. **Figure 1-supplement 3.** Method for measuring the ratio of nucleus to total cell TDP-43 YFP from mean pixel intensity of MBNs. **Figure 2-supplement 1.** Mushroom body lobes (MBLs) show age-related, region-specific TDP-43^G298S^ cytoplasmic accumulation and axonal fragmentation. **Figure 4-supplement 1.** TDP-43^G298S^ overexpression in MBNs reduces arousal, increasing day and night sleep. **Figure 5-supplement 1.** Mutant TDP-43 overexpression in MBNs is sufficient to reduce lifespan. **Figure 6-supplement 1**. mRNAs enriched with TDP-43^G298S^ overexpression in *Drosophila* MBs. **Figure 6-supplement 2.** Functional annotation of enriched targets in fly models of TDP-43 driven dementia. **Figure 7-supplement 1.** Dally-like protein is a target of TDP-43^G298S^ in MBNs. **Figure 7-supplement 2.** TDP-43^WT^ protein expression is not reduced by the presence of a second UAS-driven transgene. **Figure 7-supplement 3.** TDP-43^WT^ YFP expression levels is not reduced by concomitant expression of a second UAS-driven transgene (mCD8 RFP).

**Additional file 2:** **Table S1E** Nucleus to whole cell TDP-43 YFP ratios summarized in Figure 1e. **Table S1F** Summary statistics for cell number data presented in Figure 1f. **Table S2A** Summary statistics for mCD8RFP intensity presented in Figure 2a. **Table S2B** Summary statistics for YFP particle size values presented in Figure 2b. **Table S3** Summary statistics for y-maze data presented in Figure 3. **Table S4A** Summary statistics for night sleep presented in Figure 4a. **Table S4B** Summary statistics for sleep bout length presented in Figure 4b. **Table S4C** Summary statistics for sleep bout number presented in Figure 4c. **Table S6A** Wild-type TDP-43 GOterms from differential gene expression presented in Figure 6. **Table S6B** Mutant TDP-43 GOterms from differential gene expression presented in Figure 6. **Table S7CD** Y-maze summary statistics for data presented in Figures 7c and 7d. **Table S8** mRNAs enriched in nuclei with TDP-43 associated cryptic exons. **Table S9** Summary of phenotypes observed in wild-type and mutant fly models.

## Data Availability

The RNA-seq datasets supporting the conclusions of this article are available in the NCBI GEO Bioproject GSE217213. The datasets supporting the conclusions of this article are included within the article. Additional files are available from ScholarSphere (doi:10.26207/jq6p-w169), and are cataloged in the Key Resources table. Please refer to the included readme file for variable descriptions. Summary statistics for each figure can be found in the Additional file 2.

## Abbreviations

AD: Alzeheimer’s disease
ALS: Amyotrophic lateral sclerosis
BMP: Bone morphogenic protein
CSF: Cerebrospinal fluid
DAMs: Drosophila activity monitors
FDR: False discovery rate
FTD: Frontotemporal dementia
GPI: Glycosyl phosphatidyl inositol
HSPG: Heparin sulfate proteoglycans
MBs: Mushroom bodies
MBLs: Mushroom body lobes
MBNs: Mushroom body neurons
MNs: Motor neurons
TDP-43: TAR DNA binding Protein 43
