# Supplementary material for: Modelling TDP-43 proteinopathy in Drosophila uncovers shared and neuron-specific targets across ALS and FTD relevant circuits
Source: Acta Neuropathol Commun. 2023 Oct 20;11:168. doi: 10.1186/s40478-023-01656-0 (PMC10588218; doi:10.1186/s40478-023-01656-0)
Supplement: Supplementary file 1 — Additional file 1: Figure 1-supplement 1. Maximum intensity projections showing split Gal4 driver line SS01276 expression in the 3rd instar larva. Figure 1-supplement 2. TDP-43G298S localization in MBN cell bodies. Figure 1-supplement 3. Method for measuring the ratio of nucleus to total cell TDP-43 YFP from mean pixel intensity of MBNs. Figure 2-supplement 1. Mushroom body lobes (MBLs) show age-related, region-specific TDP-43G298S cytoplasmic accumulation and axonal fragmentation. Figure 4-supplement 1. TDP-43G298S overexpression in MBNs reduces arousal, increasing day and night sleep. Figure 5-supplement 1. Mutant TDP-43 overexpression in MBNs is sufficient to reduce lifespan. Figure 6-supplement 1. mRNAs enriched with TDP-43G298S overexpression in Drosophila MBs. Figure 6-supplement 2. Functional annotation of enriched targets in fly models of TDP-43 driven dementia. Figure 7-supplement 1. Dally-like protein is a target of TDP-43G298S in MBNs. Figure 7-supplement 2. TDP-43WT protein expression is not reduced by the presence of a second UAS-driven transgene. Figure 7-supplement 3. TDP-43WT YFP expression levels is not reduced by concomitant expression of a second UAS-driven transgene (mCD8 RFP). [file 40478_2023_1656_MOESM1_ESM.docx]

**SUPPLEMENTAL INFORMATION**

**AUTHORS**: R Keating Godfrey^1,2*^, Eric Alsop^3^, Reed T Bjork^1^, Brijesh S Chauhan^4^, Hillary C Ruvalcaba^1^, Jerry Antone^3^, Lauren M Gittings^5^, Allison F Michael^1^, Christi Williams^1^, Grace Hala’ufia^1^, Alexander D Blythe ^1^, Megan Hall^3^, Rita Sattler^5^, Kendall Van Keuren-Jensen^3^, Daniela C Zarnescu ^1,4,^*

**AFFILIATIONS**:

^1^Department of Molecular and Cellular Biology, 10007 E. Lowell St, Life Sciences South, University of Arizona, Tucson AZ 85721, USA

^2^ current address: McGuire Center for Lepidoptera and Biodiversity, Florida Museum of Natural History, 3215 Hull Road, University of Florida, Gainesville, FL 32611

^3^ Translational Genomics Research Institute, 445 N 5^th^ St, Phoenix, AZ 85004, USA

^4^ Cellular and Molecular Physiology, 500 University Drive

Crescent Building C4605, Penn State Medical Center, Hershey PA 17033, USA

^5^ Dept. of Translational Neuroscience, Barrow Neurological Institute, 350 W Thomas Road, Phoenix, AZ 85013, USA

* corresponding authors: Daniela C Zarnescu [dcz102@psu.edu](mailto:dcz102@psu.edu), R Keating Godfrey [rkgodfrey@floridamuseum.ufl.edu](mailto:rkgodfrey@floridamuseum.ufl.edu)

Key Resources

| REAGENT or RESOURCE | SOURCE | IDENTIFIER |
| --- | --- | --- |
| Antibodies | | |
| Anti-GFP-FITC | Rockland | Cat#: 600-402-215 |
| Anti-Fasciclin-II (ID4) | DSHB | RRID: AB_528235 |
| ChromoTek GFP-Trap® Magnetic Agarose | Proteintech | Cat#: gtma  RRID: AB_2631358 |
| Dally-like (13G8) | DSHB | RRID: AB_528191 |
| Polyclonal Alexa Fluor-568-conjugated goat anti-mouse secondary | ThermoFisher Scientific | Cat#A-11004;  RRID: AB_2534072 |
| Polyclonal Alexa Fluor-647-conjugated goat anti-mouse secondary | ThermoFisher Scientific | Cat#A-21235;  RRID: AB_2535804 |
| Living Color mouse Ab anti-GFP | Cell Signaling Technology | Cat# 2955 |
| Rabbit Ab beta-actin | Cell Signalling Technology | Cat#4967S |
| IRDye 800CW goat anti-mouse | LI-COR | Lot#D21115-25 |
| IRDye 680RD goat anti-rabbit | LI-COR | Lot# D21207-05 |
| Deposited data | | |
| Figure_1D_RatioAnalysis.csv | ScholarSphere | doi:10.26207/jq6p-w169 |
| Figure_1F_CellCountsAnalysis.csv | ScholarSphere | doi:10.26207/jq6p-w169 |
| Figure_2C_FluorescenceIntensity_RFP.csv | ScholarSphere | doi:10.26207/jq6p-w169 |
| Figure_2D_TDPYFPParticleSize.csv | ScholarSphere | doi:10.26207/jq6p-w169 |
| Figure_3_Y_maze.csv | ScholarSphere | doi:10.26207/jq6p-w169 |
| Figure_4A_4B_Day_Night_Sleep_SourceData.csv | ScholarSphere | doi:10.26207/jq6p-w169 |
| Figure_4C_SleepBoutLength_SourceData.csv | ScholarSphere | doi:10.26207/jq6p-w169 |
| Figure_4C_SleepBoutNumber_SourceData.csv | ScholarSphere | doi:10.26207/jq6p-w169 |
| Figure_5_Survival_2022_SourceData.csv | ScholarSphere | doi:10.26207/jq6p-w169 |
| Figure_6A_Targets_SourceData.csv | ScholarSphere | doi:10.26207/jq6p-w169 |
| Figure_6B_6C_MB_MN_overlap_SourceData.csv | ScholarSphere | doi:10.26207/jq6p-w169 |
| Figure_7C_7D_DlpOE_YMazeSourceData.csv | ScholarSphere | doi:10.26207/jq6p-w169 |
| NCBI GEO Bioproject GSE217213 | NCBI |  |
| Experimental models: Organisms/strains | | |
| *D. melanogaster*:  -p65ADZp in attP40/ CyO; ZpGdbd  in attP2 (MB-specific expression) | Yoshi Aso | *SS01276* |
| *D. melanogaster*: Oregon-R ; ;  (OR-R, working memory assays) | Todd Schlenke | Oregon-R |
| *D. melanogaster*:  OR-R; UAS-TDP-43^WT^::YFP  (working memory assays) | Shaun Davis (Schlenke laboratory), constructed from Estes et al., 2011; Estes et al.; 2013 |  |
| *D. melanogaster*: w^1118^ | Bloomington *Drosophila*  Stock Center | RRID:BDSC_5905 |
| *D. melanogaster*: w^1118^ ; UAS-TDP-43^WT^ (untagged TDP-43 for sleep assays) | J. Paul Taylor (Ritson et al. 2020) |  |
| *D. melanogaster*: w^1118^ ; ; UAS-TDP-43^G298S^ (untagged TDP-43 for sleep assays) | Takeshi Iwatsubo (Ihara et al. 2013) |  |
| *D. melanogaster*: w^1118^ ; UAS-TDP-43^WT^::YFP; UAS mCD8::RFP (MB morphology and IPs) | Robert Kraft (Zarnescu laboratory) |  |
| *D. melanogaster*: w^1118^ ; UAS-TDP-43^G298S^::YFP UAS-mCD8::RFP (MB morphology and IPs) | Robert Kraft (Zarnescu laboratory) |  |
| w^1118^ ; UAS-mCD8::RFP (control) |  | BL 27391 |
| y w^1118^ ; ; UAS-YFP (control) |  | BL 6660 |
| Software | | |
| R | The R Foundation for Statistical Computing  (2021) | 4.1.2 (2021-11-01) -- "Bird Hippie" |
| RStudio | RStudio Team (2021) | RStudio 2021.09.0+351 "Ghost Orchid" Release |

Tables

Summary tables in file Godfrey_et_al_Supplemental_Tables.xlsx provide summary statistics or interpretive data corresponding to the following figures and the corresponding supplemental:

Table_S1E_NucCellRatio Figure 1e

Table_S1F_CellNumber Figure 1f

Table_S2a_mCD8RFP_Intensity Figure 2a

Table_S2B_YFP_ParticleSize Figure 2b

Table_S3_Ymaze Figure 3

Table_S4A_DayNightSleep Figure 4a

Table_S4B_SleepBoutLength Figure 4b

Table S4C_SleepBoutNum Figure 4c

Table_S6A_WT_MBOnly_GO_Term Figure 6a

Table_6B_G298S_MBOnly_GO_Term Figure 6b

Table_S7C_S7D_DlpYmaze Figure 7c and 7d

Table_S8_snRNA_FTD_patients Figure 8

Table_S9_Model_comparisons

Supplemental Figures


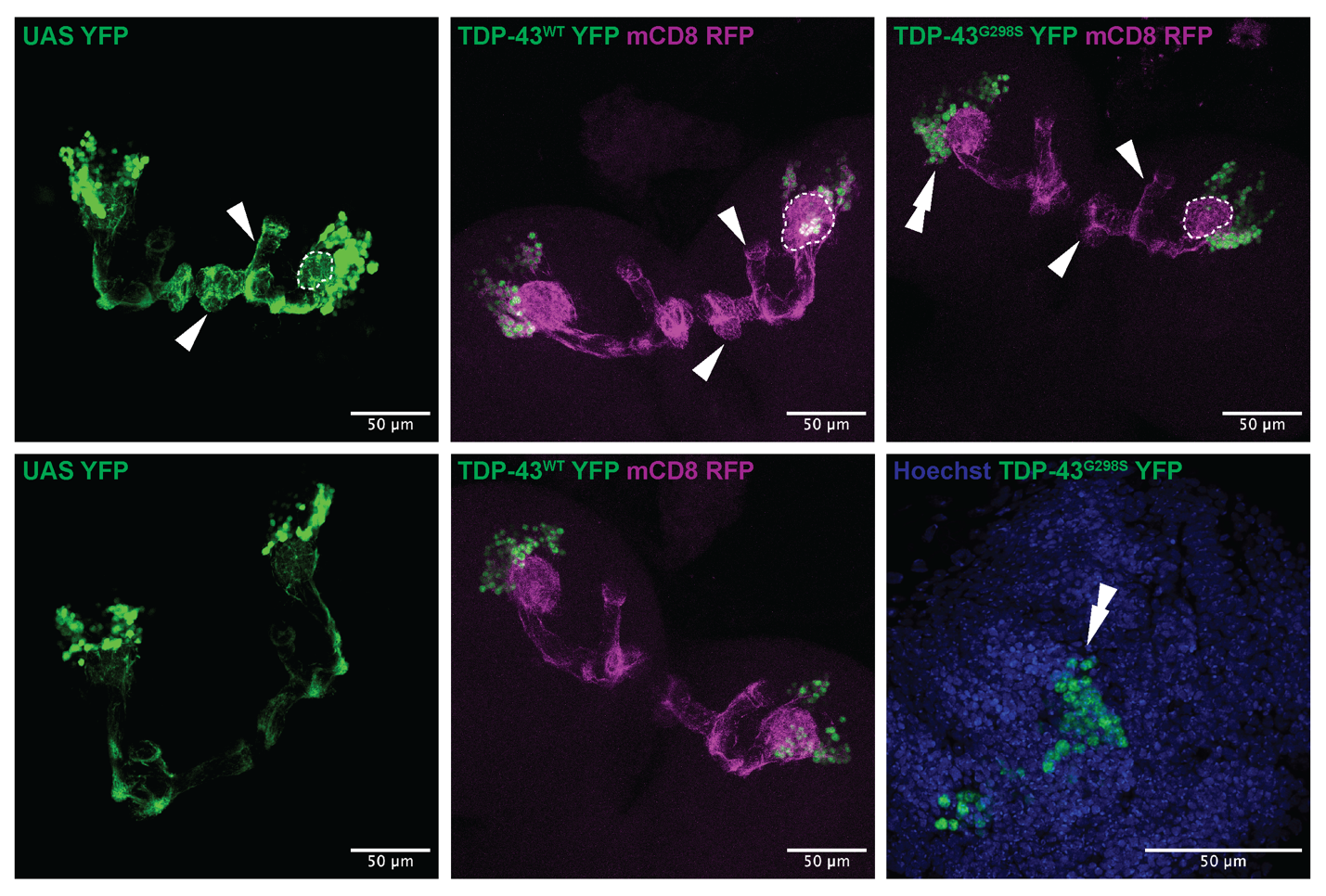


Figure 1-supplement 1. Maximum intensity projections showing split Gal4 driver line *SS01276* expression in 3^rd^ instar larva. YFP (left) labels cell bodies, calyx (dendrites, dotted outline), and lobes (axons, arrows). In TDP-43 YFP mCD8 RFP brains, TDP-43 YFP is restricted to cell bodies (middle and right) while mCD8-RFP allows visualization of all cell membranes. Measurement of TDP-43 YFP nucleus to whole cell ratio was performed from high magnification (100X) images of the calyx region (example lower left with double arrows corresponding to region in lower magnification image above).


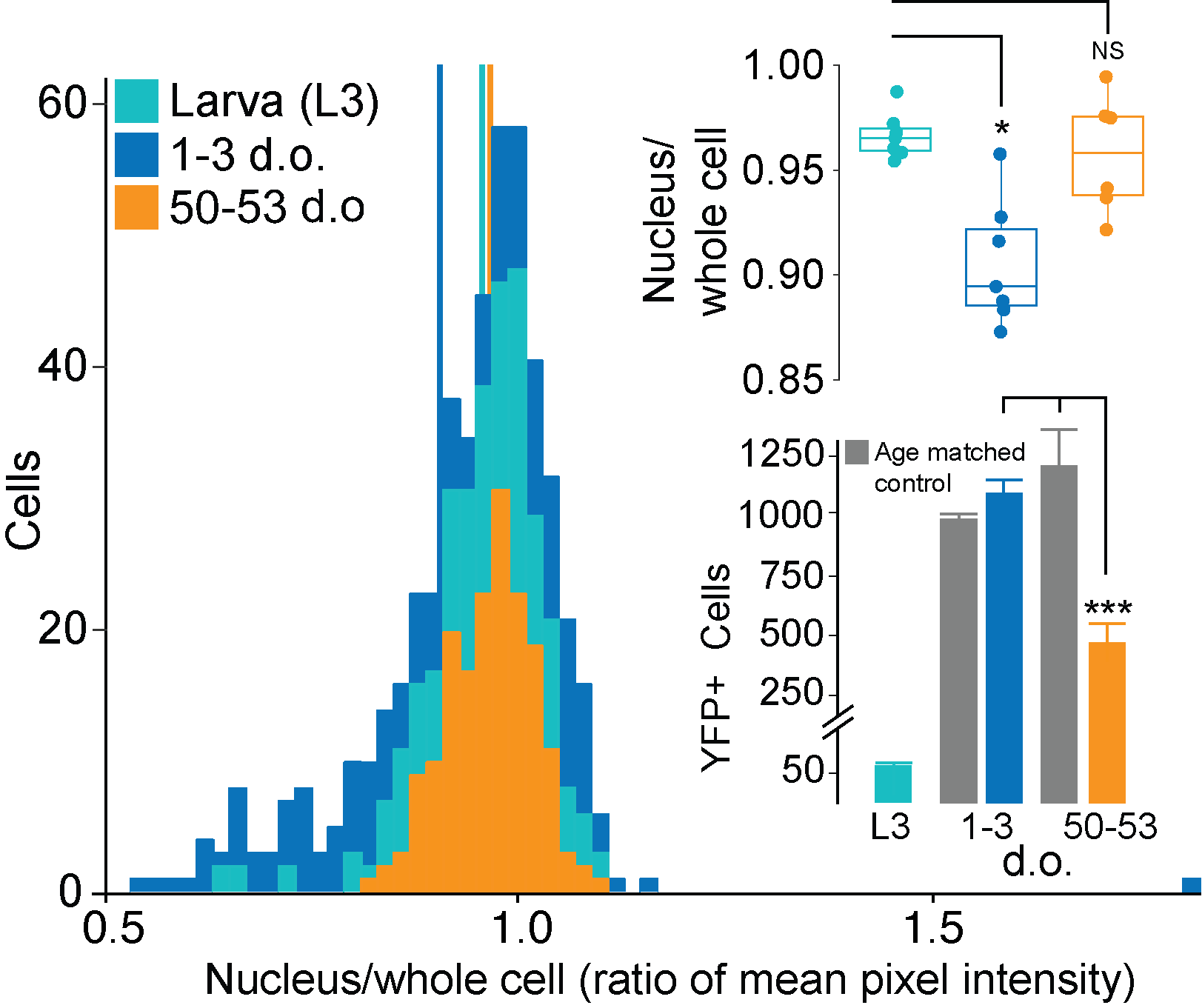


Figure 1-supplement 2. TDP-43^G298S^ localization in MBN cell bodies. Histograms showing distribution of TDP-43 signal intensity ratios (cell nuclei to total cell) shift with age (days old, d.o.). Boxplot depicting age-specific TDP-43^G298S^ signal intensity ratios. * = P < 0.05.

Figure 1-supplement 3. Method for measuring the ratio of nucleus to total cell TDP-43 YFP from mean pixel intensity of MBNs. White box on left depicts area of insets on right. Top, right inset shows cell body perimeter traced and measured in TDP-43 YFP channel (= total cell mean pixel intensity), middle and lower right panel show nuclear boundary traced in Hoechst channel and measured in TDP-43 YFP channel (= nucleus mean pixel intensity). Ratio of nucleus to total cell used for quantification of nuclear depletion in MBNs with age.


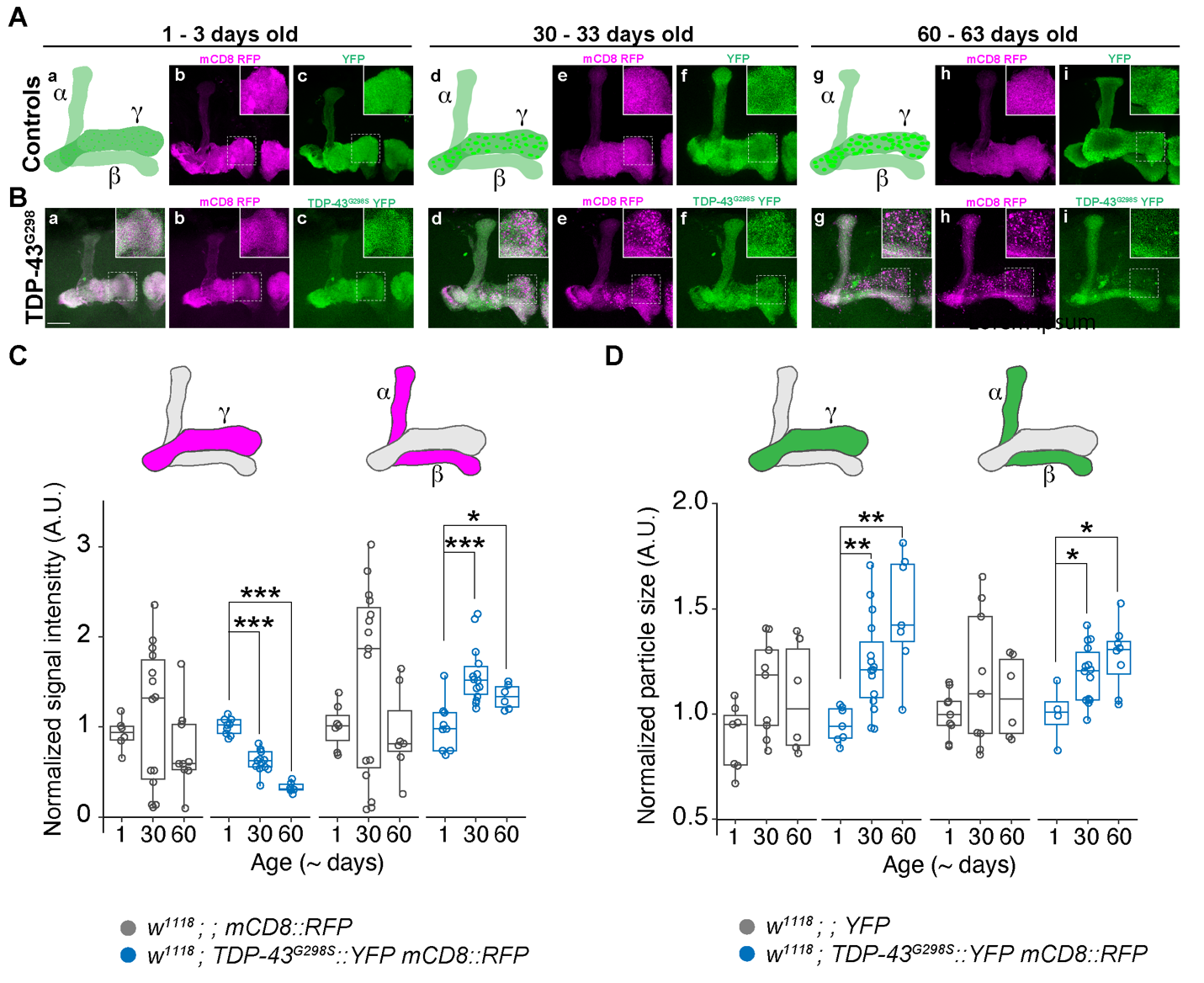


Figure 2-supplement 1. Mushroom body lobes (MBLs) show age-related, region-specific TDP-43^G298S^ cytoplasmic accumulation and axonal fragmentation. (A) Illustrations of changes in MBLs targeted by TDP-43 OE over time (a, d, g) alongside morphology of young (b, c,) middle-aged (e, f) and old (h, i) control flies expressing membrane-bound RFP or cytoplasmic YFP. (B) Over expression of TDP-43^G298S^ in MBNs results in axonal localization of TDP-43 in young adult flies (c) and dystrophic neurites in middle-aged (e) and old (h) flies. (C) Signal intensity of RFP with age in γ and α/β lobes. (D) Changes in YFP particle size with age in γ and α/β lobes.


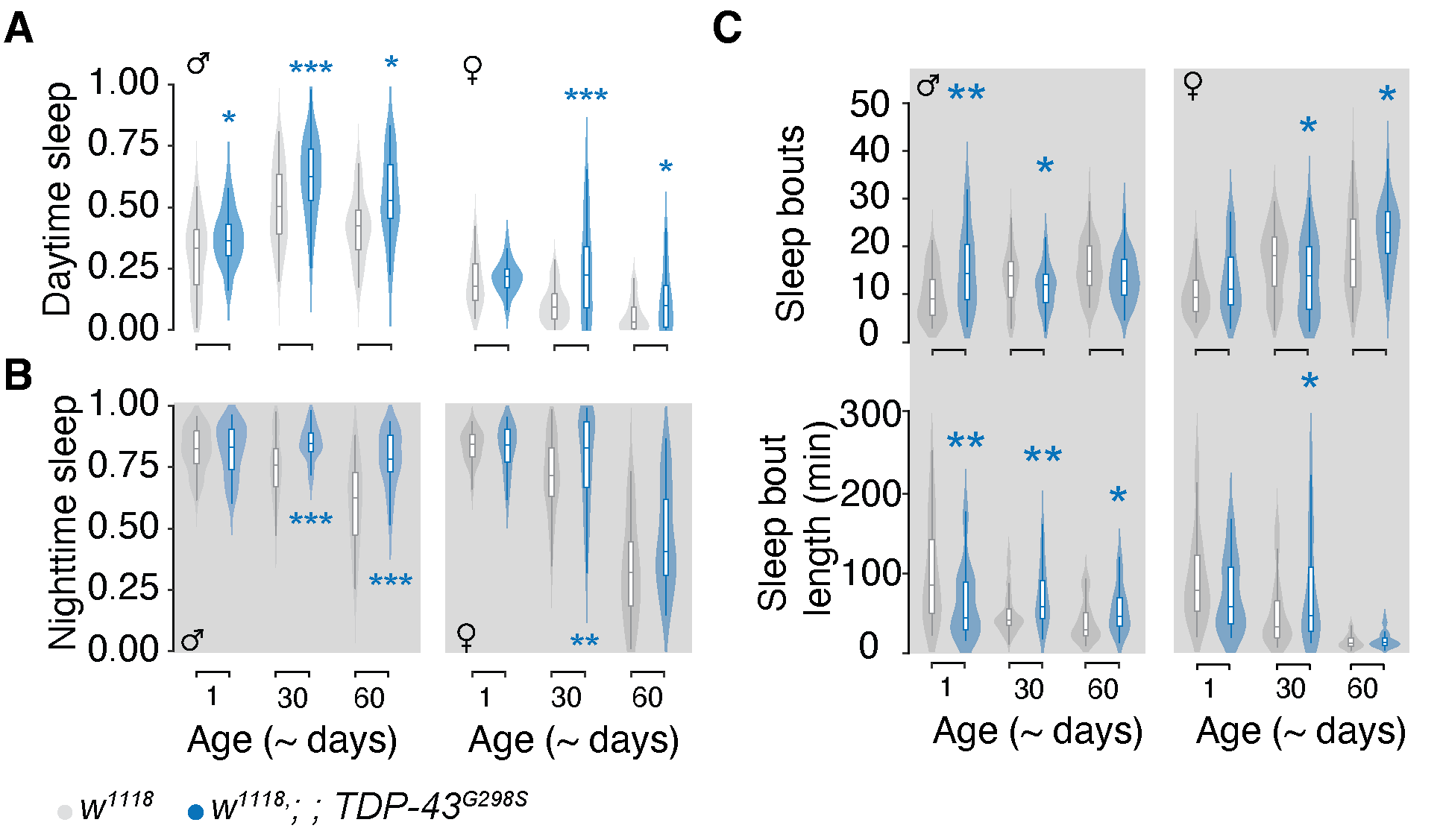


Figure 4-supplement 1. TDP-43^G298S^ overexpression in MBNs reduces arousal, increasing day and night sleep. (A) Proportion of time flies spent sleeping during the day and (B) at night. (C) Sleep fragmentation assessed by number of sleep bouts (top panel) and mean bout length (bottom panel) during the night. Male data on the left and female data on the right in each panel. * = P <0.05; ** = P < 0.01; *** = P < 0.001.


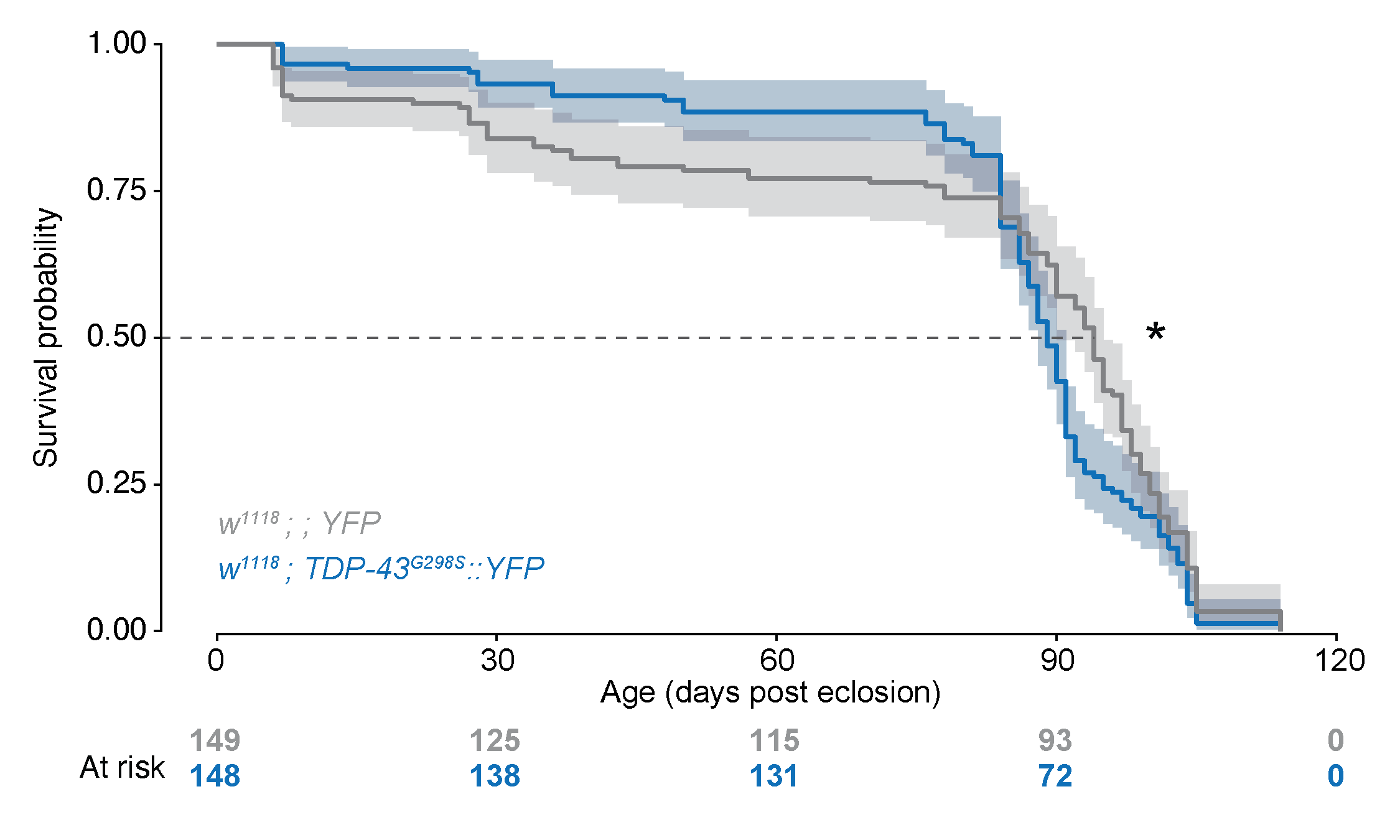


Figure 5-supplement 1. Mutant TDP-43 overexpression in MBNs is sufficient to reduce lifespan. Data from male and female flies pooled for analysis. * P < 0.05.


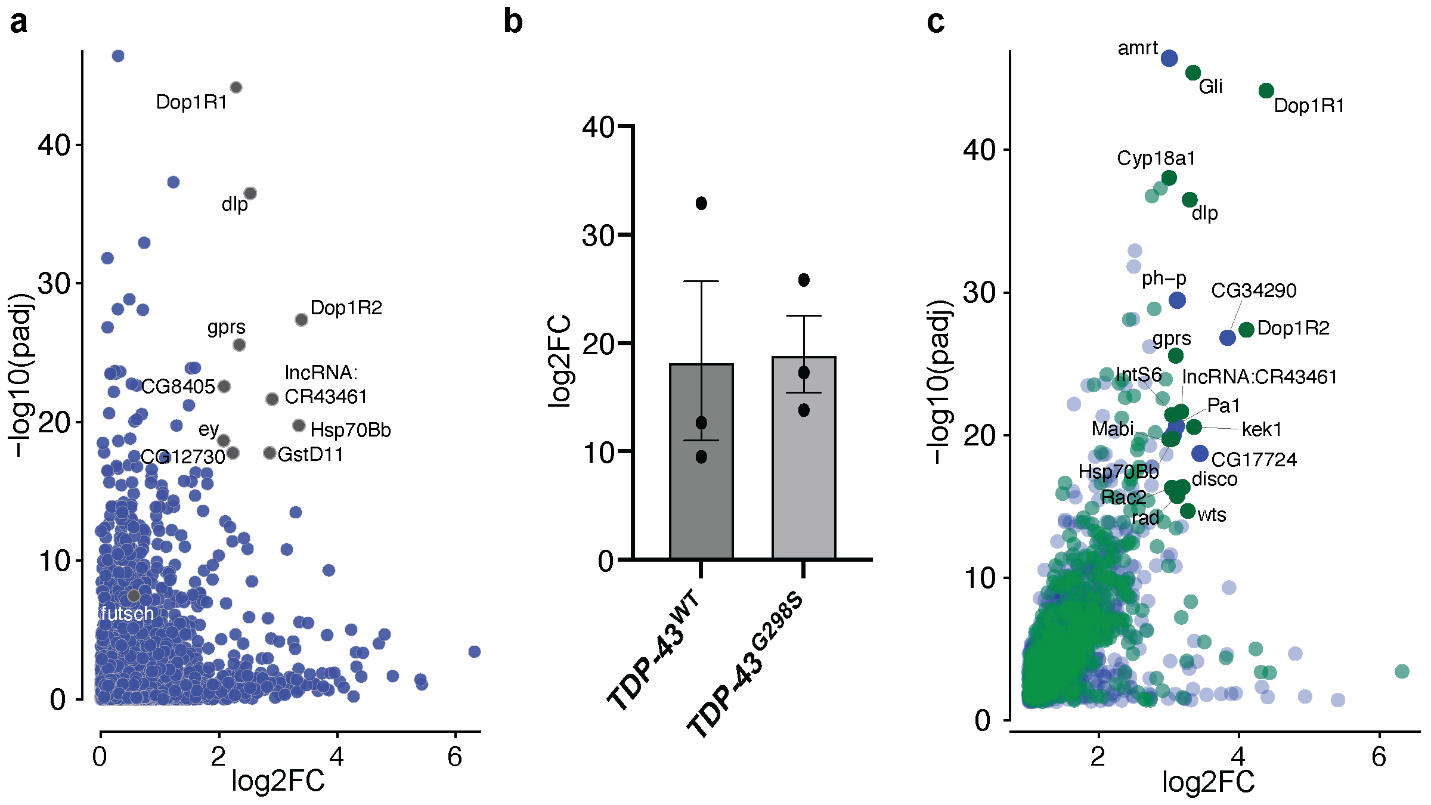


Figure 6-supplement 1. mRNAs enriched with TDP-43^G298S^ overexpression in a *Drosophila*  MBs. (A) Volcano plot displaying enriched mRNAs. Y-axis depicts Log2 Fold Change after subtraction of YFP control values. Blue circles indicate log2 fold change > 2 and P < 1 x 10^-14^. (B) qPCR validation of *dlp* enrichment in TDP-43 complexes immunoprecipitated from adult heads expressing TDP-43 with *SS01276*. (C) Volcano plot displaying mRNAs enriched in TDP-43^G298S^ complexes that are MB-specific (blue) or shared between MB and MN models; saturated green circles indicate shared targets that show log2 Fold Change > 2 and P < 1 x 10^-14^; blue circles indicate MB targets with log2 fold change > 3 and P < 1 x 10^-14^.


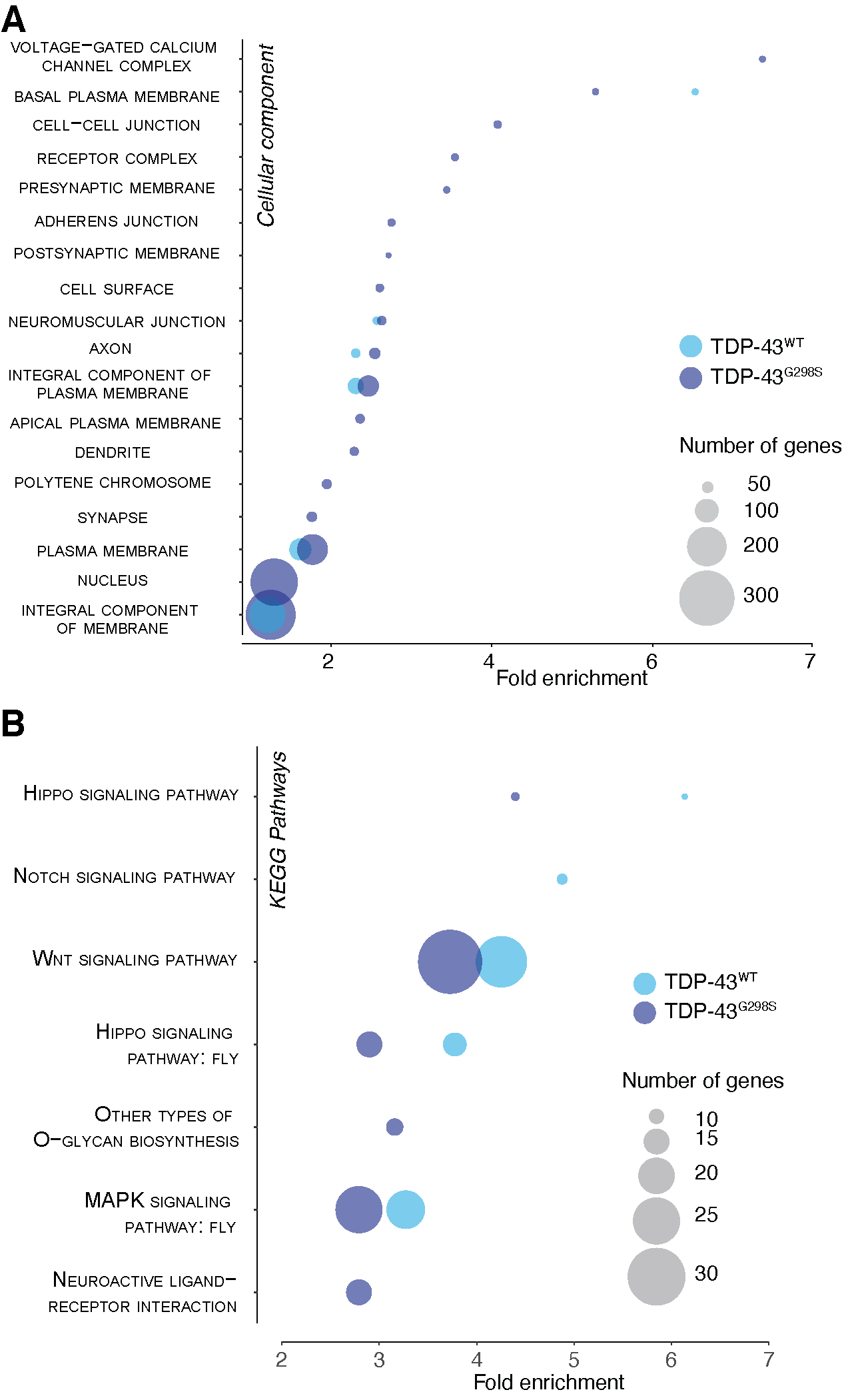


Figure 6-supplement 2. Functional annotation of enriched targets in fly models of TDP-43 driven dementia. (A) Cellular component of genes enriched in each TDP-43 model ranked by fold enrichment over all identified mRNAs in fly heads. (B) KEGG pathway analysis of targets enriched in each TDP-43 model ranked by fold enrichment over all identified mRNAs in fly heads.


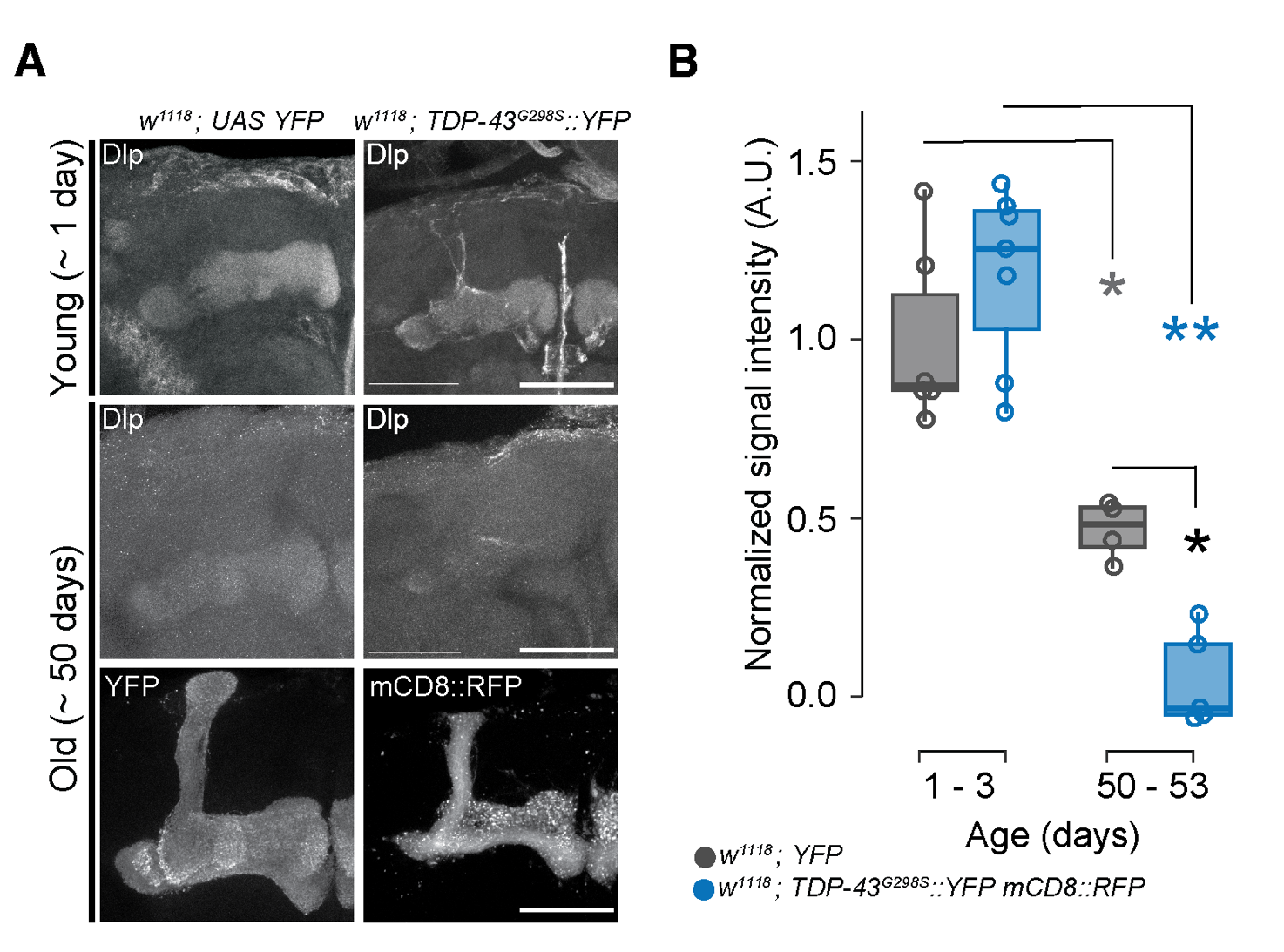


Figure 7-supplement 1. Dally-like protein is a target of TDP-43^G298S^ in MBNs where it mediates TDP-43 dependent working memory deficits.. Deficits in working memory are evident in young adults. (A) Age-dependent loss of Dlp antibody labelling in mushroom bodies. Cytoplasmic YFP (Control) or mCD8 RFP (*TDP-43^G298S^*) in aged flies indicating intact MBLs show decreased Dlp signal at ~ 50 days. Colored asterisks indicate statistical comparisons by age within a genotype. (B) Change in Dlp signal intensity in MBLs with age. Scale bar = 50 μm. * = P < 0.05, ** = P < 0 .01.


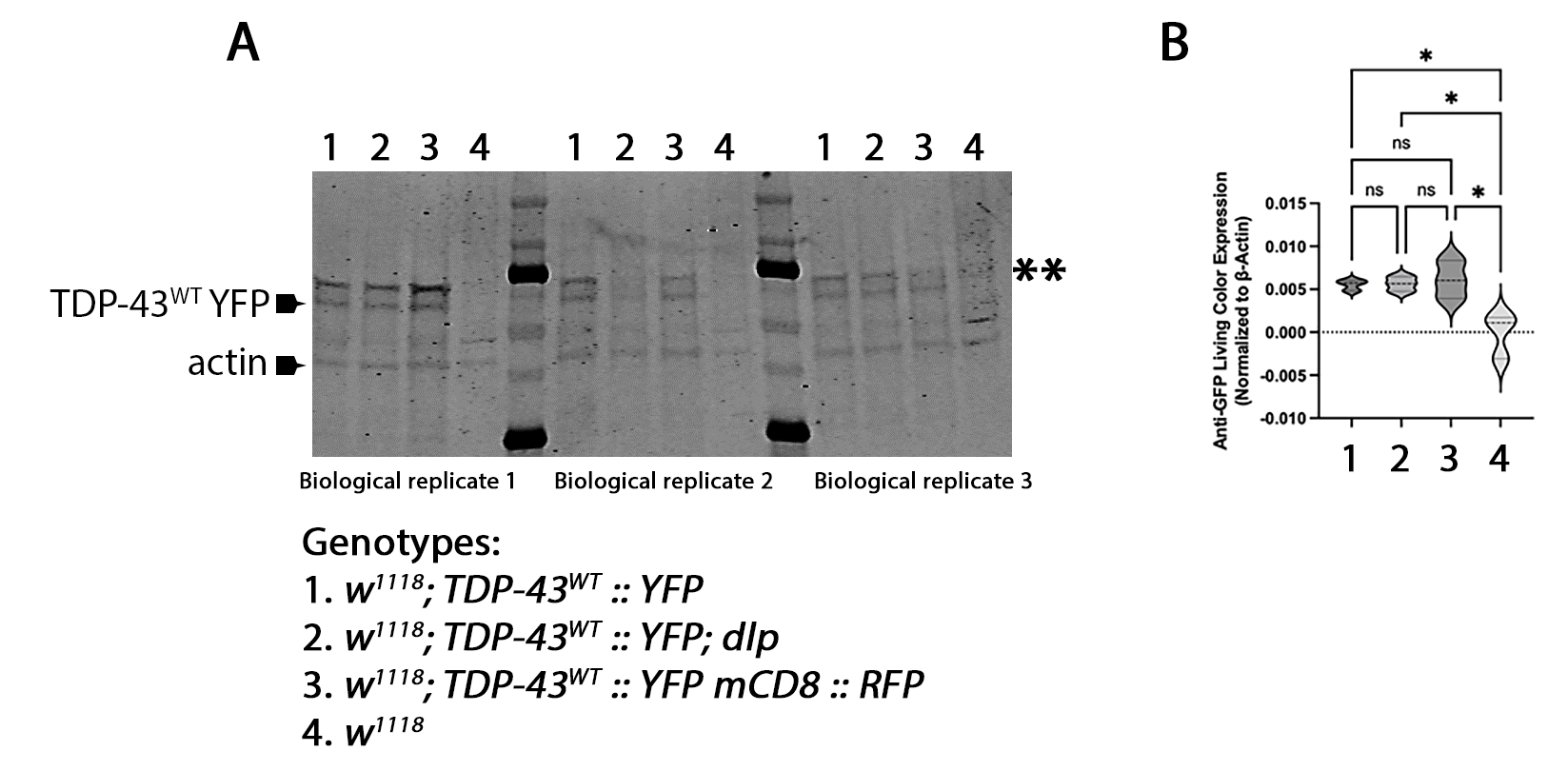


Figure 7-supplement 2. TDP-43^WT^ protein expression is not reduced by the presence of a second UAS-driven transgene. (A) Western blot showing TDP^WT^-43 YFP expression driven by the mushroom body driver line, *SS01276* in three different genotypes: *TDP-43^WT^::YFP*, *TDP-43^WT^::YFP dlp* *OE*, and *TDP-43^WT^::YFP mCD8::RFP*. *w^1118^* controls were used to confirm antibody specificity against GFP/YFP in fly tissues. TDP-43 YFP and actin, as indicated by arrows, left side. Double asterisk, right side, indicates non-specific band. (B) Quantification of western blot in (A). Genotypes and replicates, as indicated.


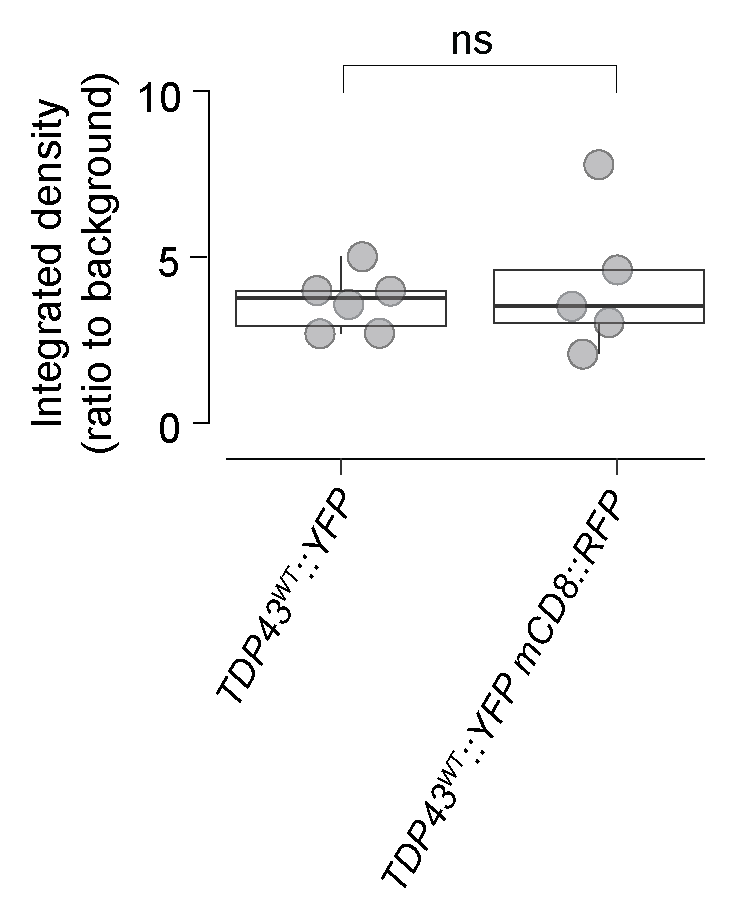


Figure 7-supplement 3. TDP-43^WT^ YFP expression levels upon concomitant expression of a second UAS-driven transgene (mCD8 RFP). TDP^WT^-43 YFP expression driven by the Split Gal4 mushroom body driver line, *SS01276* in two genotypes: *OR; TDP-43^WT^::YFP* and *w^1118^; TDP-43^WT^::YFP mCD8::RFP*. Means compared using Wilcoxon Rank Sum.
